# Pink noise in dyads’ semantic similarity trajectories predicts conversation enjoyment

**DOI:** 10.1126/sciadv.aeb2684

**Published:** 2026-11-06

**Authors:** Kathryn C. O’Nell, Kiara L. Sanchez, Emily S. Finn

**Affiliations:** Department of Psychological and Brain Sciences, Dartmouth College, Hanover, NH 03755, USA.

## Abstract

Conversation is a core component of human social life. Many influential theories treat conversation as a linear system, i.e., the sum of its parts. However, linear accounts of semantic entrainment lose explanatory power at their upper limits: Discussing perfectly aligned opinions becomes boring. We propose an alternate account that considers the multiscale organization of semantic similarity over the course of a conversation, as indexed by pink noise. Here, we test whether pink noise can be found in interlocutors’ conavigated trajectories through semantic space. Not only is pink noise present, but its strength predicts positive conversation outcomes like enjoyment and connection, increases with practice, and is reflected in a large language model’s representation of conversation quality. These results demonstrate an important role for scale-free dynamics in characterizing successful conversation.

## INTRODUCTION

Good conversation is like pornography—it is difficult to define, but we know it when we see it. Despite the difficulty of the task, building a mechanistic understanding of how good conversation emerges is essential to the study of human social interaction. Existing work takes several approaches: investigations based on high-level folk-psychological constructs (*1*–*3*), data-driven approaches that objectively quantify low- and mid-level features of conversation (*4*–*7*), and the application of domain-general cognitive mechanisms to the specific task of conversation (*8*–*11*). While this work has made some inroads into the thorny problem of characterizing good conversation, many of these approaches rely primarily on linear methods that assume conversation to be the sum of its parts (i.e., individual speakers and speech acts) rather than an interactive dynamical system (*12*).

While natural conversation is composed of many verbal and nonverbal signals, we focus on semantics—i.e., the actual words people use—as a particularly important channel for communication. Prior work has demonstrated that the degree to which conversation partners use similar language predicts outcomes ranging from romantic interest and relationship stability (*13*) to group cohesion (*14*). Much of this body of work, however, operationalizes similarity as a global property of the conversation—computed as an average or aggregate score—rather than a dynamic signal, leaving open the questions of (i) how semantic similarity fluctuates over the course of a conversation and (ii) how those fluctuations shape outcomes. Some past work indicates that contextual and temporal variability in semantic similarity matter for outcomes, suggesting that conversation contains dynamics that aggregate measures cannot fully capture (*15*–*20*). Hence, shifting to an explicitly dynamical approach can help us better understand how conversation works.

Characterizing how semantic similarity fluctuates over time means asking not just how much it varies but how variation at different timescales is related—whether rapid, turn-to-turn shifts are organized together with the slow arc of the conversation. No measure computed at a single, fixed timescale can answer this, since coupling is a property of the relationship between scales rather than of any single scale. We therefore ask whether semantic similarity exhibits structure that recurs across timescales rather than concentrating at any one, a property captured by a signal’s noise color. Pink noise, sometimes called “1/*f* noise,” occurs in signals where power is inversely proportional to frequency on a log-log scale, such that lower frequencies show higher power. These signals sit halfway between purely stochastic white noise, with equal power in all frequencies, and Brownian drift, where lower frequencies dominate.

To build intuition about how pink noise and its alternatives might function in conversation, we can imagine hypothetical conversations that differ in the noise color of their semantic similarity trajectories. In a conversation exhibiting pink noise, interlocutors would not stay perfectly aligned forever, nor would they drift apart randomly. Instead, they would remain aligned for a while, diverge as one introduces something new, then realign as the other picks it up and develops it further. This pattern—periods of stability nested within periods of change across many timescales—produces pink noise, a “goldilocks zone” between chaos and rigidity. In a conversation closer to white noise, however, interlocutors’ alignment varies unpredictably from each turn to the next: They might briefly align because one responds directly to the other, but this alignment is just as likely to disappear immediately, replaced by an unrelated comment or abrupt shift in direction. Red noise would bring a conversation toward the other extreme: When partners are closely aligned, they remain aligned for long stretches, continuing to repeat, elaborate, or restate the same ideas. When they diverge, they remain apart, with limited reconnection.

These three possible patterns do not differ in how similar partners are on average over the course of a conversation, but rather in how that similarity evolves over time. White noise reflects similarity that fluctuates without temporal structure, suggesting limited sustained coordination. Red noise reflects overly persistent similarity, suggesting rigid conversational dynamics. Pink noise reflects an intermediate regime in which similarity shows both persistence and change across multiple timescales, consistent with a system that balances stability and flexibility.

Here, we demonstrate that interlocutors’ negotiations in semantic space exhibit nontrivial multiscale structure and that the strength of the pink noise signal predicts positive conversational outcomes like enjoyment and connection. We find evidence for this in two independent dyadic conversation datasets, in addition to naturally occurring sources of data like two-host podcasts and speed-dating encounters. We also demonstrate that large language models (LLMs) asked to generate enjoyable conversations produce scripts with stronger pink noise signals, suggesting that this relationship between enjoyment and multiscale organization is encoded in the large quantity of human-generated text used to train LLMs.

## RESULTS

### Pink noise emerges in semantic similarity dynamics during conversation

Our first goal was a proof of concept: to demonstrate that pink noise in interlocutors’ movement toward and away from each other in semantic space is present in conversation. For this, we used data from the CANDOR corpus (*5*), a dataset consisting of 1656 open-ended conversations between strangers [mean conversation length in minutes = 34.1 (SD = 9.2); mean conversation length in turns = 320.2 (SD = 92.4)]. For each conversation, the transcript was split into turns, each turn was embedded in high-dimensional semantic space, and the distance between adjacent turns was calculated to produce a semantic similarity trajectory for the conversation (Fig. 1; see Materials and Methods for more details). For each semantic similarity trajectory, detrended fluctuation analysis (DFA) (*21*, *22*) was used to determine a noise scaling coefficient for the conversation. A coefficient of 0.5 represents white noise, while a coefficient of 1.0 represents pink noise. Within the CANDOR corpus, 94% of conversations had noise scaling coefficients greater than 0.5 (mean = 0.62, SD = 0.08; Fig. 2A), suggesting that these conversations exhibit some degree of scale-free dynamics in their semantic similarity trajectories.

**Fig. 1.**
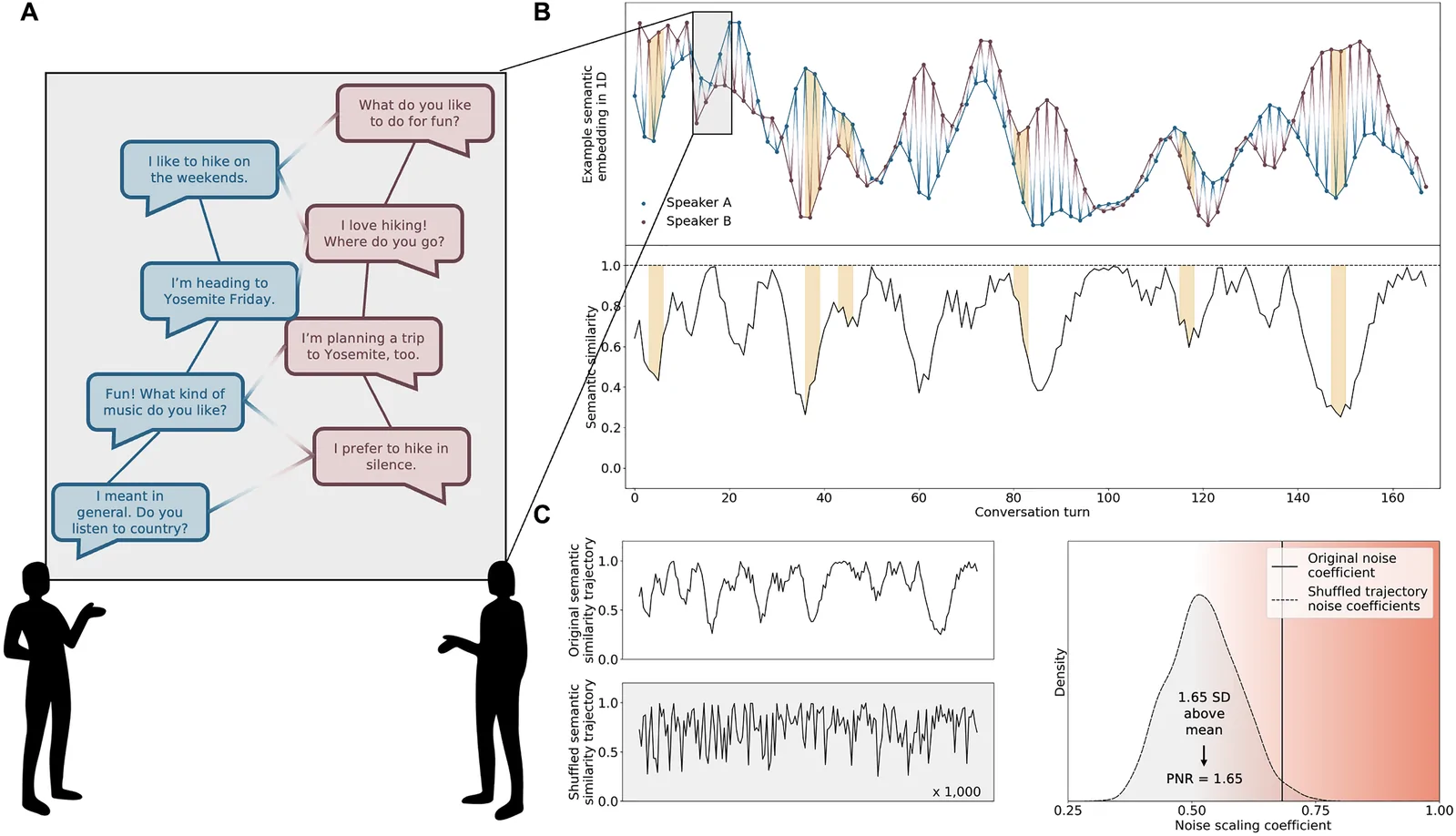
Methods overview. (**A**) An artificial conversation snippet. (**B**) A depiction of how a semantic similarity trajectory is generated for a single conversation transcript. Top: Each point represents the 1-dimensional projection of a 768-dimensional semantic embedding of a conversational turn; see the expanded box for an example. The red and blue lines represent each of the two speakers’ separate trajectories through semantic space, and the gradient lines connecting each adjacent turn represent the (cosine) distance between these turns’ embeddings. Bottom: A depiction of the whole conversation’s semantic similarity trajectory. Note that the yellow bars connecting points on this trajectory to the maximum value of 1 are the same length as the yellow bars in the top of this figure, which represent semantic distance between embeddings of adjacent turns. (**C**) A depiction of how a semantic similarity trajectory’s PNR is calculated. Left top: The example semantic similarity trajectory from (B), which has a noise scaling coefficient of 0.71. Left bottom: An example similarity trajectory generated by shuffling the signal in the panel above. A null distribution of noise scaling coefficients is built for each conversation by repeating this shuffling process 1000 times and calculating a noise scaling coefficient for each of these shuffled trajectories. Right: A depiction of a conversation’s original noise scaling coefficient as compared to its null distribution. A conversation’s PNR is the *z*-score of the original noise scaling coefficient compared to its null.

**Fig. 2.**
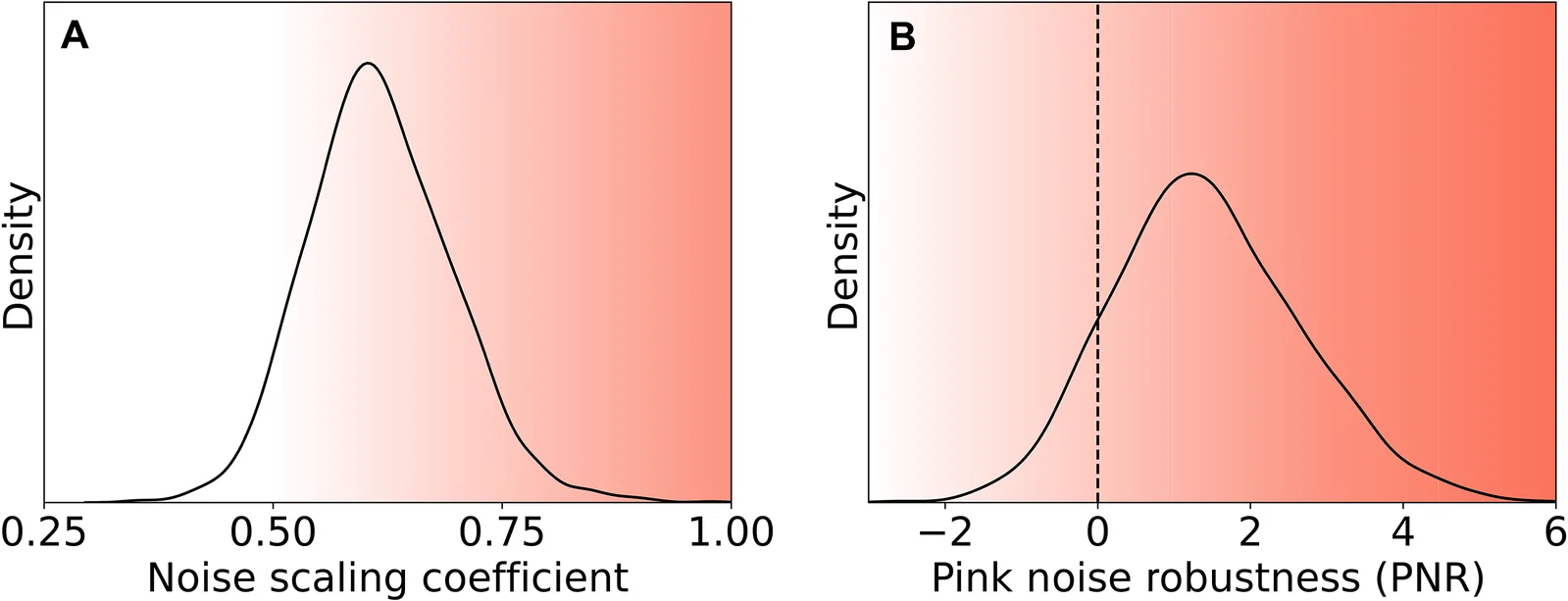
Distribution of noise scaling coefficients and PNR scores for the CANDOR corpus. (**A**) For semantic similarity trajectories of conversations in the CANDOR corpus (*N* = 1656), noise scaling coefficients fell between 0.35 and 0.98, with a mean of 0.62 (SD = 0.08), indicating the presence of pink noise. (The scaling coefficient for pure white noise is 0.5, while the scaling coefficient for pure pink noise is 1.0.) (**B**) For semantic similarity trajectories in the CANDOR corpus, the mean PNR score was 1.43 (SD = 1.24), indicating that the pink noise present in these conversations generally exceeds that predicted by conversation-level null distributions.

While signal noise color is a size-invariant measure, two complications can arise when working with shorter signals (i.e., those with approximately 300 or fewer observations). First, fewer opportunities to observe power at low-frequency bands can result in systematically lower (whiter) noise scaling coefficients. On the other hand, short stochastic signals produce a broader distribution of scaling coefficients than their longer counterparts, raising the likelihood of a spuriously high (pinker) scaling coefficient. To account for these two sources of bias, we transformed the raw noise scaling coefficients into *z*-scores by comparing them to a conversation-specific null generated by calculating the scaling coefficients for 1000 shuffled semantic similarity trajectories (see Materials and Methods for more details). The CANDOR conversations had a mean *z*-scored noise scaling coefficient of 1.43 (SD = 1.24; Fig. 2B), confirming that observed noise scaling coefficients above 0.5 were not merely artifacts of signal length. In the remainder of our analyses, we use these *z*-scores, which we henceforth term “pink noise robustness (PNR)” scores, rather than raw scaling coefficients, as they provide a more unbiased index of the degree of pink noise present in a conversation.

As an additional robustness check to confirm that the observed pink noise in these conversations’ semantic similarity trajectories is rooted in the original order of turns, not just temporally agnostic distributional statistics, we recalculated PNR after shuffling the order of turns in each conversation. This maintains turn structure but disrupts the conversational flow by which each turn builds on the one(s) before it. As expected in a signal with multiscale organization, the original semantic similarity signals had significantly higher PNRs than their modified counterparts (paired *t* test: *t* = −15.2, *P* < 0.001; fig. S3, left).

Overall, we found that the conversations in the CANDOR dataset demonstrate a tendency toward pink noise en masse but that there is also considerable variability in PNRs across conversations. This variation is useful because it allows us to test whether the strength of the pink noise signal in a conversation predicts positive conversational outcomes. In the following sections, we explore the dyadic nature of multiscale organization in conversation and then relate PNR to various outcome measures across conversations.

#### Pink noise reflects dyadic structure beyond individual contributions

Attributing multiscale structure to the dyad, rather than to its constituent interlocutors, is inherently difficult. Individuals continuously shape and are shaped by their shared interactional context, so individual and dyadic contributions cannot be cleanly partitioned. This separability problem imposes a scope constraint on our claims: We might naively predict that interleaving turns drawn from different conversations would result in whiter conversations, but the trajectories resulting from this procedure (see Materials and Methods for more details) exhibit higher PNRs than either the individual or dyadic signals from the conversations from which their turns were drawn (both *P* < 0.001; see Materials and Methods for full model specification). However, these high PNR scores do not arise from genuine multiscale coordination, but rather as an artifact: Coarse, conversation-level structure is preserved while turn-to-turn similarity scores are compressed toward zero. Interleaving conversations drops the mean semantic similarity value per conversation from 0.19 to 0.15 (paired *t* test: *t* = −41.3, *P* < 0.001), while the SD decreases from 0.15 to 0.14 (*t* = −13.9, *P* < 0.001). This compression suppresses high-frequency fluctuations, thereby inflating relatively low-frequency power (see fig. S1 for more details). Since pink noise can be produced degenerately, we limit our claims about the dyadic nature of multiscale structure specifically to realized dyads, comparing multiscale structure in individual and dyadic semantic similarity trajectories across multiple manipulations.

Across realized conversations, several independent lines of evidence converge on the dyad as the most relevant unit for analyzing multiscale structure in conversation. First, individual-level PNRs are significantly lower than their dyadic counterparts (see Supplementary Models details, Model 1.0.1). In other words, when individual speakers’ turns are removed from their dyadic context, their semantic similarity trajectories shift toward white noise. Second, PNR does not behave like a stable individual trait. For speakers who participated in multiple conversations, their individual-conversation PNRs (PNR scores derived for a single participant’s turns in a conversation) were no more similar to their individual PNRs in other conversations than to others’ PNRs (Mantel test *P* = 0.25; fig. S7; see the “CANDOR corpus” section in Materials and Methods for full details). Third, the multiscale structures of dyadic and individual trajectories respond differently to perturbations of turn-taking structure. For all trajectories—both individual and dyadic—we recalculated PNR after permuting the location of turn breaks within the conversation. This transformation maintains the original order of words, number of turns, and distribution of turn lengths, disrupting only the coordinative give-and-take of the conversation. In dyadic conversations, this shuffling procedure significantly reduces the PNR of the signal (paired *t* test; *t* = 13.3, *P* < 0.001; fig. S3, middle). This could be attributable to disruptions in turn-handoff structure, or, more parsimoniously, just the rechunking of the embedded semantic units. In the latter case, however, one would expect individuals’ semantic similarity trajectories to experience the same decrease in PNR after undergoing the same shuffling procedure, which they do not. Individual semantic similarity trajectories exhibit slightly higher PNRs after this shuffling procedure (paired *t* test; *t* = −4.0, *P* < 0.001; fig S3, right).

We interpret this pattern through the framework of interaction dominance, under which system-level dynamics cannot be decomposed into independent component contributions. So, to further test whether dyadic PNR is reducible to individual PNRs beyond the linear case, we regressed dyadic PNR on a flexible symmetric function of the two individual PNRs—symmetric so that both interlocutors enter identically—combining the pair’s mean, their squared difference, an interaction between the two, and a cubic term of the mean (see Supplementary Models, Model 1.0.2). This model accounted for only 1.4% of the variance in dyadic PNR, indicating that dyadic PNR captures temporal structure not captured by its individual counterparts. While this is not definitive proof of interaction dominance, it more strongly licenses an interpretation of dyadic PNR as a genuinely emergent dyadic measure rather than a symmetric recombination of individual-level pink noise.

#### Interaction dominance and metastable states in dyadic trajectories

Interaction-dominant coordination further predicts metastability: flexible switching between states of a system rather than random movement between states or permanent stability in a fixed regime. We operationalized these states by partitioning each conversation’s semantic-similarity trajectory into high- and low-variance segments via a median split on local variance (see the “Testing the dyadic nature of multiscale organization” section in Materials and Methods). The choice of a median split for two regimes is arbitrary, but metastability makes a falsifiable prediction: Regimes defined by one statistical property should systematically differ in other properties as well. In this case, they do: High-variance regimes are also high-mean regimes (see Supplementary Models, Model 1.1.1). If pink noise reflects the conversational system’s movement between these regimes, then disrupting the temporal organization of the regimes should degrade PNR. We altered regime-shifting in two complementary ways (see the “Testing whether disrupting interactive properties of conversation reduces pink noise” section in the Supplementary Materials; figs. S4 and S5): moving all instances of a particular state to be concurrent (regime-sorted) and interleaving such that the signal moves between states more rapidly (regime-interleaved; see fig. S3 for a graphical depiction of this procedure). Both manipulations significantly reduced PNR relative to the original trajectories (paired *t* tests | regime-sorted: *t* = −40.4, *P* < 0.001; regime-interleaved: *t* = −15.9, *P* < 0.001).

Critically, these regimes are dyadically defined rather than inherited from either speaker: Individually derived regimes do not align with their dyadic counterparts above chance levels (*P* = 0.21; see Supplementary Models, Model 1.1.2). We additionally tested whether individual and dyadic variance trajectories were correlated, in case the median split method for defining regimes was obscuring overlapping state transitions. However, they were weakly anticorrelated (*P* = 0.015; see Supplementary Models, Model 1.1.3).

Together, these results start to converge on an interaction-dominant, dyad-centric account of pink noise in semantic similarity. The interpretation remains open, and we do not claim that dyadic structure is wholly independent of the individuals who generate it; but across multiple, mutually reinforcing tests, the dyad—rather than solely the individual—emerges as an appropriate level at which to understand this structure.

### Pink noise predicts enjoyment and sense of connection during conversation

Having established that movement through semantic space in CANDOR conversations demonstrated varying degrees of pink noise, we then sought to test whether the strength of these pink noise signals was associated with positive conversation outcomes. In the CANDOR dataset, participants in each conversation answered extensive pre- and postconversation surveys. We sorted the 205 items that elicited numerical responses into six categories: those related to (i) enjoyment, (ii) connection, (iii) engagement and memory, (iv) demographic information, (v) one’s own personality traits, and (vi) one’s perception of their partner’s personality traits.

Using these survey results, we took two approaches to relating pink noise to conversation success. First, in a mass univariate approach, we correlated PNR with each of these 205 variables across conversations. After Bonferroni multiple hypothesis correction, seven outcome variables were significantly positively correlated with PNR: how enjoyable participants found the conversation, how much longer a participant would have continued talking past the end, how much a speaker disclosed, how much a participant believed their partner disclosed, a participant’s consistent desire to remain in the conversation rather than ending early, how much longer a participant believed their partner would have liked to keep talking, and how long the conversation lasted beyond the required 25 min. With a more lenient Benjamini/Hochberg false discovery rate (FDR) multiple hypothesis correction, the number of outcome variables significantly correlated with PNR increased to 44, 22 of which were enjoyment related, and 8 of which were connection related (Fig. 3A). Both enjoyment- and connection-related variables were vastly overrepresented in the sets that survived multiple hypothesis correction (*chi*-*square* | Bonferroni version: *P* < 0.001; FDR version: *P* < 0.001).

**Fig. 3.**
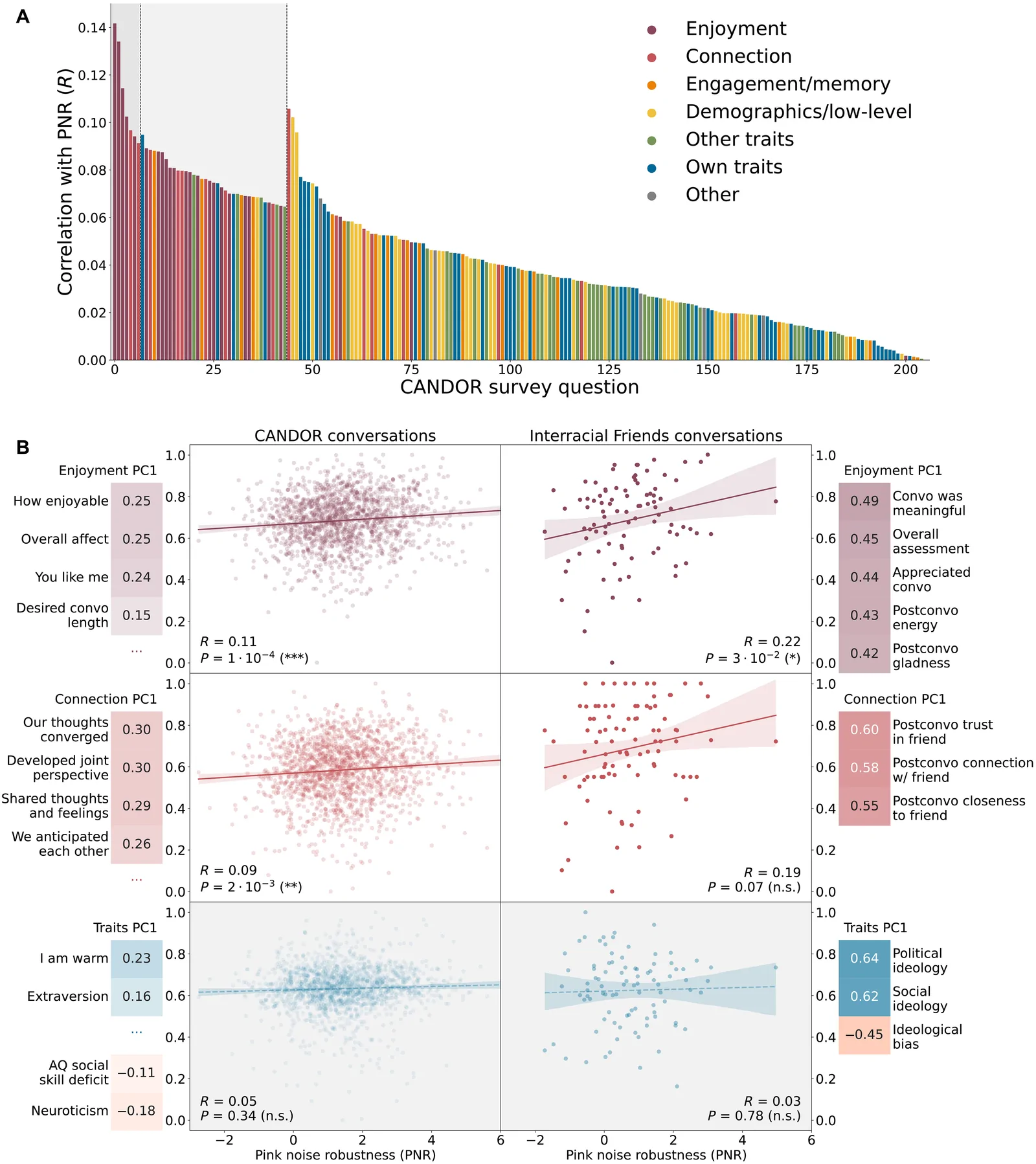
PNR predicts positive conversation outcomes in two corpora. (**A**) Results from a mass-univariate analysis correlating PNR scores with each of the 205 numerical variables in the CANDOR survey across the 1656 conversations in the dataset. Variables are sorted along the *x* axis by statistical significance (*P* value), from more significant (left) to less significant (right) and colored by question category. After Bonferroni correction for multiple comparisons, seven variables were significantly correlated with PNR (dark gray background), and after Benjamini/Hochberg FDR correction, 44 variables were significantly correlated with PNR (light gray background). Enjoyment- and connection-related variables were vastly overrepresented in these sets of variables (Bonferroni version: *P* < 0.001; FDR version: *P* < 0.001). Note that some of the survey variables that did not survive multiple hypothesis correction have higher correlation coefficients than those that did survive correction; this is because different questions in the CANDOR survey had different response rates, leading to different degrees of freedom across the correlations. (**B**) PNR is correlated with the first principal component of variables related to enjoyment (top) and connection (middle), but not individual traits (bottom) in CANDOR (left) conversations. The same holds true for enjoyment, though not connection, in the Interracial Friends (right) conversations (*N* = 97). While each category (enjoyment, connection, and individual traits) is constructed from different sets of questions in the CANDOR and Interracial Friends corpora, they capture comparable conceptual dimensions. Correlation statistics in the CANDOR subplot are reported as Bonferroni-corrected *P* values, as we tested PNR against each outcome variable with no a priori hypothesis. The Interracial Friends analyses were performed as explicit replications of the CANDOR analyses, so these are not corrected for number of correlations tested. Significance per Pearson correlation is indicated as follows: *P < 0.05, **P < 0.01, ***P < 0.001. n.s., not significant.

Given that many survey variables were highly collinear with one another, especially within categories, we additionally tested whether conversation PNR was correlated with a summary measure of each category (i.e., score on the first principal component from a principal components analysis performed on all variables from that category). First principal component scores for enjoyment and connection were significantly correlated with PNR after multiple hypothesis correction (correlation coefficient *R* = 0.11 and 0.09; corrected *P* < 0.001 and 0.002; Fig. 3B, left), while this was not true for the rest of the categories (engagement/memory: *R* = 0.06, corrected *P* = 0.09; demographics: *R* = 0.06, corrected *P* = 0.1; own traits: *R* = 0.05, corrected *P* = 0.3; partner’s traits: *R* = 0.05, corrected *P* = 0.2).

#### Controlling for basic conversation properties

These relationships were robust to low-level covariates: Even when we add predictor terms for turn duration (mean and SD), turn word count (mean and SD), backchannel rate, transcription quality proxy measures, and conversation length (turn count and duration in seconds) as competing variables in a linear model, PNR still accounts for unique explanatory variance toward enjoyment and connection (see Supplementary Models, Models 2.1.1 and 2.1.2).

This pattern of results suggests that a higher conversational PNR predicts positive outcomes like enjoyment and connection. However, this does not necessarily demonstrate that a multiscale approach outperforms simpler linear methods. While the nonlinear nature of these signals (see the “Testing the nonlinearity of semantic similarity trajectories” section in Supplementary Text for more details) would suggest a benefit from multiscale analysis, we performed a series of controls to test whether a more parsimonious linear account could account for the same variance in positive outcome variables.

#### Controlling for static and simple linear measures of similarity

We tested whether outcomes could be predicted from the average semantic similarity between pairs of adjacent turns across the whole conversation (a static measure), the variance in similarity across pairs of turns (a close inverse analog of time-locked covariation in semantic space: A value of 0 would indicate that speakers kept a constant, rigid distance in semantic space between them), and the slope of the similarity trajectory over time (simple synchronization, without a role for multiscale fluctuation). In multiple regression models predicting conversation enjoyment and connection based on these three variables and PNR, all but average similarity were significant predictors (see Supplementary Models, Models 2.2.1 and 2.2.2). These results suggest that the multiscale view afforded by pink noise captures variance in positive conversation outcomes not fully accounted for by simpler linear metrics.

#### Controlling for more complex linear measures

In addition, we tested whether PNR outperformed more complex linear measures: autoregressive moving average (ARMA) coefficients for similarity trajectories, dynamic time warping (DTW) synchrony metrics, and information-theoretic coupling (see the “CANDOR corpus” section in Materials and Methods for more details). Because these measures may contain overlapping information, we tested each as a covariate in a separate linear model competing with the original signal’s PNR for explanatory power toward enjoyment and connection ratings. In all three cases, the original PNR score captured additional unique variance toward enjoyment ratings, and for all but the ARMA control, PNR contributed unique variance toward connection ratings (see Supplementary Models; Models 2.3.2 to 2.3.6). These results suggest that pink noise in semantic space provides insights into positive conversational outcomes that go beyond those provided by linear approaches.

When we fit a model pitting PNR against the aggregate family of more complex linear measures, PNR is no longer a significant predictor of enjoyment or connection (see Supplementary Models; Models 2.3.7 and 2.3.8), indicating that PNR does not explain additional variance beyond these other measures. This provides a useful scope check on our claims: Our purpose is not to demonstrate that PNR provides unique information about a conversation’s semantic similarity structure beyond any combination of linear measures—rather that multiscale structure is associated with positive outcome variables. In the case of the aggregate family of alternative predictors, subcomponents of PNR are likely captured by other metrics: Fluctuations at a rapid scale are represented by the ARMA coefficients, while fluctuations at a global scale are represented by the DTW similarity measure (see Supplementary Models, Models 2.3.9 and 2.3.10). An advantage of PNR is that we capture both scales in one parsimonious measure.

#### Dyadic pink noise predicts outcomes above individual counterparts

Under an interaction-dominant framing of multiscale organization in semantic similarity trajectories, one would expect pink noise on the dyadic level to contribute more to a prediction of positive outcome variables than that present in individual turn trajectories. And this is, in fact, what we observe (see Supplementary Models, Models 2.4.1 to 2.4.4).

### Replication: Pink noise predicts enjoyment in a different conversational context

While the CANDOR corpus is large, it consists only of a specific type of conversation: casual small talk between strangers. To test whether pink noise robustly predicts positive outcomes across different conversational settings, we conducted a conceptual replication in a separate conversation dataset with different properties: the Interracial Friends corpus (*N* = 97 conversations). Instead of small talk, interlocutors share formative experiences; instead of strangers, these conversations occur between friends. As in the CANDOR corpus, participants answered postconversation survey questions asking about their experience of the conversation (i.e., enjoyment and sense of connection), as well as more general questions about demographics, traits, and predictions about their future relationships. In the Interracial Friends conversations, PNR still significantly predicted enjoyment (*R* = 0.22, *P* = 0.03) but was not significantly correlated with connection (*R* = 0.19, *P* = 0.07). The relationship with enjoyment was specific in that PNR did not predict a speaker’s individual traits (*R* = 0.03, *P* = 0.78; Fig. 3B, right).

Notably, the relationship between positive outcome variables and PNR in this corpus is not robust to the inclusion of total turn count and conversation duration in seconds as predictor terms in a linear model (see Supplementary Models; Models 3.0.1 and 3.0.2). However, this is likely due to the relationship between enjoyment and duration: Participants who were enjoying their conversation continued speaking beyond the required 10 min. When we instead include the mean and SD of turn word counts and temporal duration (in addition to a transcription quality proxy measure) as competing variables in a linear model, PNR still accounts for unique explanatory variance toward enjoyment (see Supplementary Models; Models 3.0.3 and 3.0.4). This result suggests that pink noise in semantic space is a useful framework for understanding positive conversation outcomes, especially enjoyment, across different contexts and types of conversations.

### Pink noise predicts real-world behavioral outcomes during speed dates

While surveys are an undeniably useful tool for assaying conversationalists’ experiences, they are also subject to individual differences in scale use, motivational factors, and participants’ perceptions of researchers’ goals. To mitigate these effects, we sought to test whether PNR could predict positive conversational outcomes outside of a laboratory setting. Unlike many real-world conversations, which have only indirect or unmeasurable outcomes, first dates result in a binary signal of success: whether the pair pursues a second date. We used conversations from a speed-dating podcast to test whether pink noise content in semantic similarity trajectories could predict which couples would choose to see each other again.

In the Blink Date corpus, pairs (*N* = 31) engaged in 10-min phone conversations prompted by a light-hearted question (e.g., “What is your most controversial food opinion?”). After the date, both participants privately indicated whether they would like to go on a second date with their partner and were considered a match if both indicated “yes” or “maybe.” In this dataset, successful dates had PNRs significantly greater than 0 (*t* = 2.2, *P* = 0.04) while unsuccessful dates did not (*t* = −0.8, *P* = 0.43; Fig. 4A), and successful dates had significantly higher PNRs than those that did not result in matches (*t* = 2.05, *P* = 0.02; Fig. 4A), even when conversation length, operationalized as duration in seconds and total turn count, is allowed to compete for variance (see Supplementary Models, Model 4.0.1). These results provide preliminary evidence that how a potential couple negotiates semantic space across scales, as indexed by pink noise, in their initial conversation predicts partner choice in a natural setting.

**Fig. 4.**
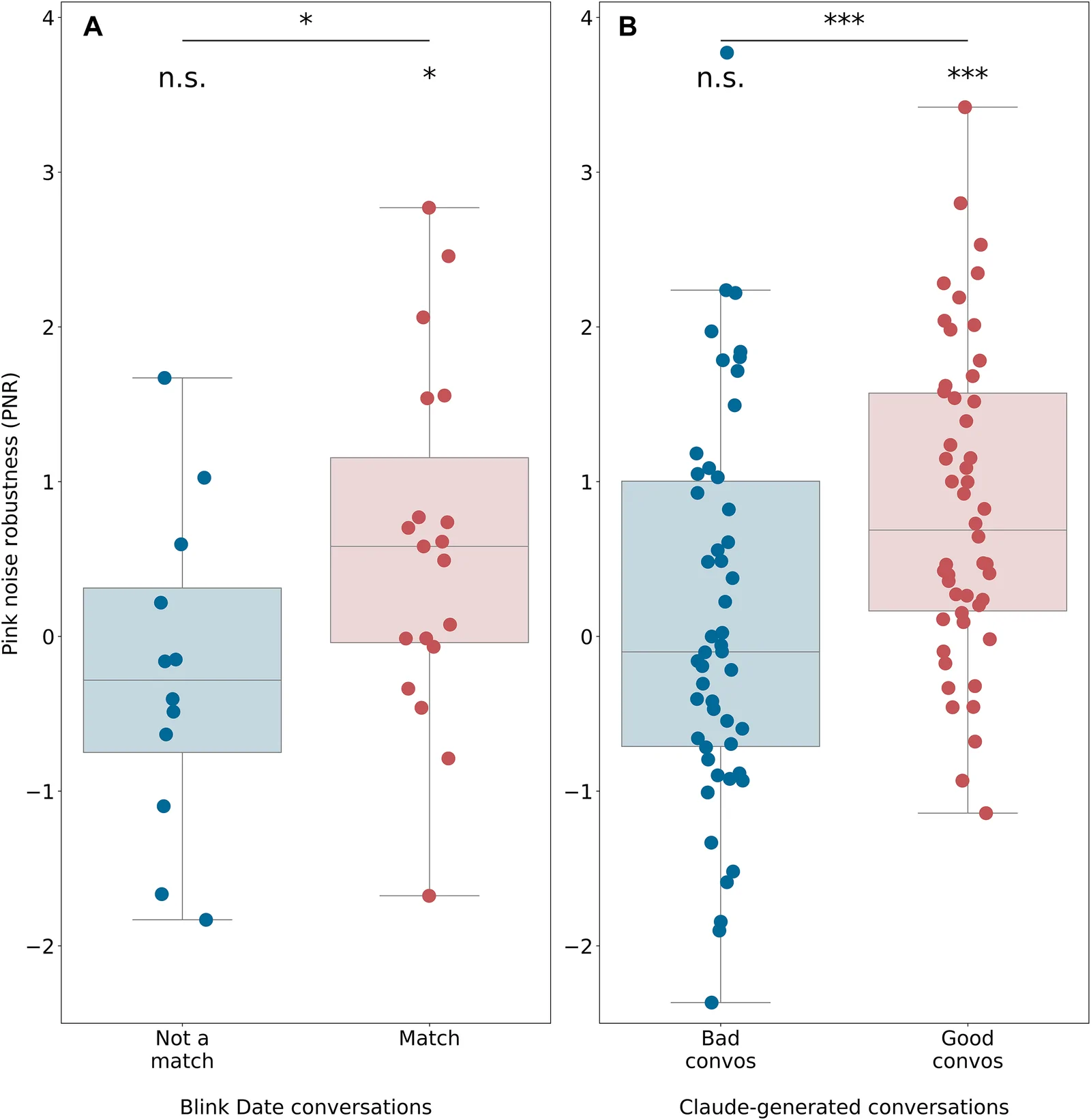
PNR predicts real-world conversation success and is present in a LLM’s representation of “good” conversation. (**A**) The distribution of PNRs for successful (match, *N* = 19) and unsuccessful (not a match, *N* = 12) first dates from the Blink Date corpus. Successful dates exhibit stronger pink noise signals than unsuccessful ones. Successful dates also exhibit PNRs significantly greater than 0, while those of unsuccessful dates do not. (**B**) Distributions of PNR scores for conversations generated by Claude Sonnet 3.7 when asked to generate high- and low- enjoyment dialogues (*N* = 50 each). The PNRs of good conversations significantly exceed 0, while those of bad conversations do not. In addition, the PNRs of good conversations significantly exceed those of bad conversations. Significance per one-sample *t* test and two-sided independent-samples *t* test are indicated as follows: *P < 0.05, **P < 0.01, ***P < 0.001.

### Pairs’ repeated conversations demonstrate stronger pink noise over time

Across various nonsocial domains, like walking gait and time estimation, pink noise signals tend to become stronger with experience. Both in the natural course of children’s development and over repeated practice trials for adults, these movements toward pink noise are hypothesized to reflect healthier coordination dynamics (*23*). Given that PNR is both predictive of positive conversational outcomes and not reducible to individual behavior, we theorized that pink noise could be a marker of healthy coordination in conversation. In this vein, we hypothesized that pairs of interlocutors who engaged in repeated conversations with each other would exhibit noise patterns that became pinker over time.

We tested this hypothesis in the natural experiment posed by the “two dudes talking” genre of podcast. Across 25 podcasts in which two hosts have relatively unconstrained conversations (see methods for full criteria), conversation PNR increased significantly across the first 50 episodes (Fig. 5), even when we include turn duration (mean and SD), turn word count (mean and SD), conversation length (turn count and duration in seconds), and transcription quality proxy measures as competing variables in a linear model (see Supplementary Models, Model 5.0.1). While this behavior is not sufficient to characterize pink noise in semantic similarity dynamics as a hallmark of optimal coordination, it is consistent with an account of multiscale behavior arising from dyadic coordination dynamics that improve with practice.

**Fig. 5.**
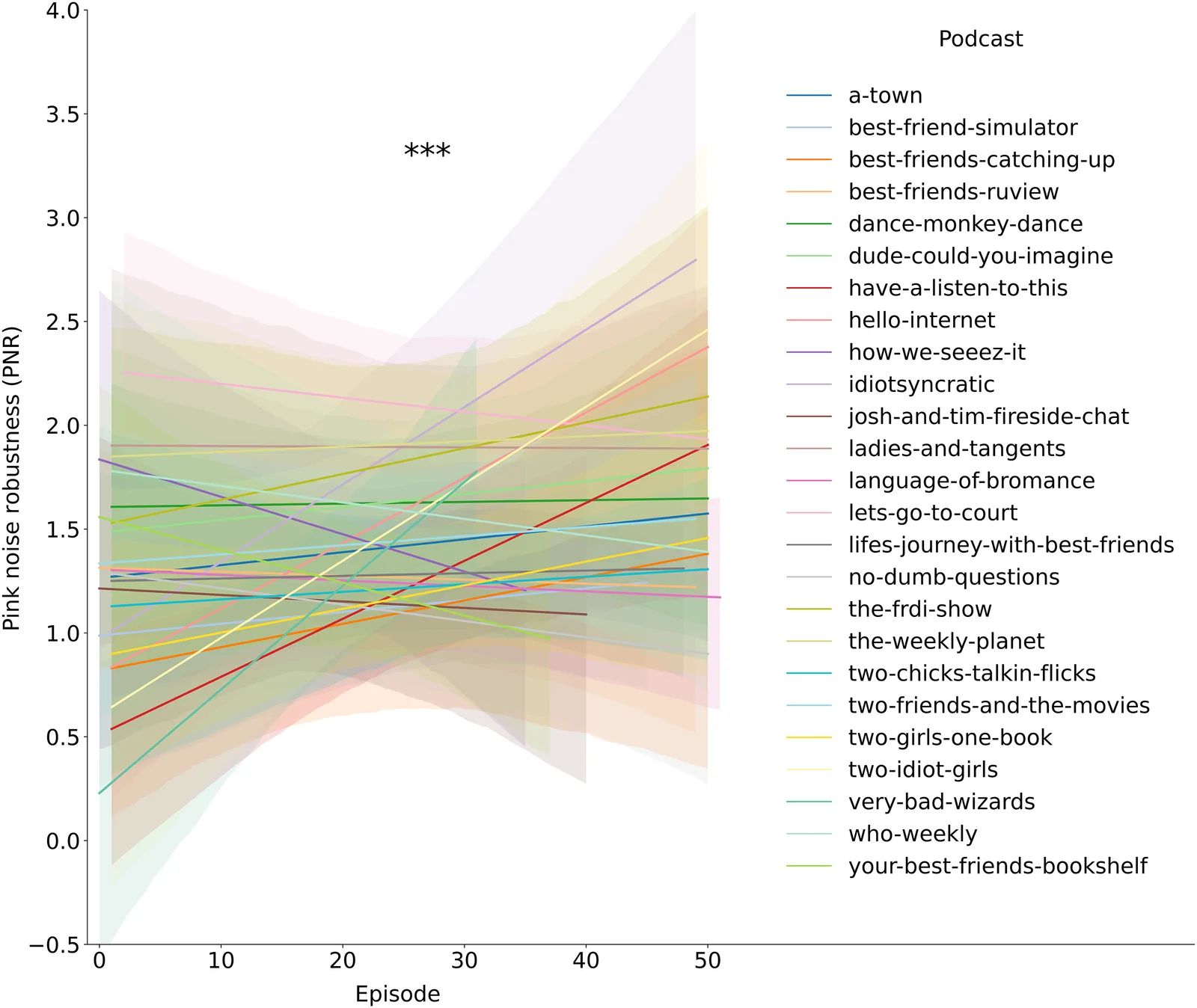
Increasing multiscale structure in semantic similarity trajectories over repeated conversations. Linear fits for the relationship between episode number and PNR for 25 dyadic conversation podcasts. Consistent with—though not demonstrative of—an account of pink noise representing healthy coordination via multiscale organization, speakers trend toward pinker noise as they gain experience conversing with their interlocutor. Significance per linear mixed-effects model of the form *PNR ∼ episode_number + (1|podcast)* is indicated as follows: *P < 0.05, **P < 0.01, ***P < 0.001.

### A LLM produces “good” conversations exhibiting pink noise

Last, we sought to test whether a LLM, Claude Sonnet 3.7 (*24*), would spontaneously produce conversations with higher PNRs when prompted to generate “good” (versus “bad”) dyadic dialogues. While there are many features that a LLM could draw upon in the creation of a “good” conversation—particularly interesting topics, or emotional arcs—if a relationship between pink noise and interaction quality exists in the large quantity of human-generated text used to train LLMs, we might expect to see that relationship mirrored in an LLM’s internal representation of conversation “goodness” regardless of the other features at play. First, we tested whether Claude could successfully modulate the perceived enjoyability of a conversation by prompting Claude to generate enjoyable versus unpleasant dialogue scripts between two strangers who meet at a coffee shop and asking online participants to rate these conversations on a scale of 1 to 5. The conversations generated by asking for enjoyable dialogues received an average score of 4.8 (SD = 0.2), while those generated by asking for unpleasant dialogues received an average score of 2.4 (SD = 0.6). The distribution-level differences were significant (*t* = 27.1, *P* < 0.001), confirming that Claude could successfully modulate the perceived enjoyability of a dialogue. Then, we tested our main hypothesis: the high-enjoyment conversations had significantly higher PNRs than their low-enjoyment counterparts (*t* = 3.2, *P* < 0.01; Fig. 4B). We additionally found that the high-enjoyment conversations had PNRs significantly greater than 0 (*t* = 5.9, *P* < 0.001; Fig. 4B) while the low-enjoyment conversations did not (*t* = 0.7, *P* = 0.50; Fig. 4B). This suggests that a system with no explicit training (to our knowledge) around the idea that enjoyable interactions are organized in accordance with distinct noise signatures has nonetheless picked up on this tendency from ingesting large amounts of human-generated text.

## DISCUSSION

Good conversation, despite its ubiquity, is notoriously difficult to define and characterize. Here, we demonstrate that the semantic similarity trajectories of natural conversations carry a nontrivial multiscale structure, as indexed by pink noise, that predicts positive outcomes like enjoyment and connection. A multiscale perspective captures what aggregate and linear approaches cannot: how fluctuations in semantic similarity are organized across a range of conversational scales, not just how similar partners are on average, or how steadily they converge. This temporal organization is not reducible to the behavior of the individual speakers and carries properties (sensitivity to turn-handoff structure and variance-based regimes) that are specific to dyadic interaction. We conceptualize these results through the theoretical lens of interaction-dominant coordination and metastability (*25*–*30*) and hope that this inspires future work to characterize conversation using these frameworks.

Our approach poses a contrast—but not necessarily a contradiction—to classical theories of linguistic entrainment. Past work has repeatedly demonstrated that interlocutors tend to converge over the course of a conversation across a variety of domains: vocal pitch (*31*), body movements (*32*), lexical properties (*33*), concept names (*34*)—even perception of one’s own personality traits (*35*). Further, these patterns of convergence, generally termed entrainment, are largely associated with better conversation outcomes. Our findings comport with this literature: A positive slope for semantic similarity over the course of a conversation is indeed predictive of conversation enjoyment and connection. However, our framework differs from entrainment in some key ways and explains additional variance in conversation enjoyment beyond single-scale measures of semantic similarity. By introducing an adaptive role for moving away from each other in semantic space, multiscale approaches solve entrainment’s asymptote problem: What happens when speakers become perfectly synchronized? On a conceptual level, this framework also allows for individual idiosyncrasies to play an important role in the emergent behavior of a conversational system instead of being attenuated by synchronization. We present this contrast not as an argument against entrainment but rather as a necessary corollary that embraces the full complexity of similarity dynamics in conversation.

The main limitation of this work is that it is observational and correlational: We take existing free-form conversations, assay the pink noise signal, and use the strength of this signal to predict positive conversational outcomes. This approach has an important strength: It suggests that nontrivial multiscale organization does emerge in natural conversation, and therefore, the predictive power it has toward positive outcomes is not limited to artificial laboratory-based conversation. However, if we want to develop a causal account, it will be necessary to build experimental tasks in which we manipulate the process of temporal organization and coordination. Our longitudinal podcast analysis gives us reason to believe that pink noise strengthens with experience but does not shed light on which aspects of experience are most important. Is this change dependent on conversing with the same interlocutor over time, or could it generalize to new conversation partners? Could we scaffold this process by explicitly teaching interlocutors about multiscale coordination or by providing live prompts when conversations enter a period dominated by low- or high-frequency shifts in semantic space? Answering these questions will be important for using our findings to improve real-world conversations. Of particular interest is the development of behavioral supports to help scaffold more comfortable conversation for people with social deficits, like those on the autism spectrum. In addition, building an interventional account of healthy coordination in conversation would allow for the development of more human-like artificial conversation agents and could set the stage for technologically mediated conversations about difficult issues, like political or racial identity.

Here, we have responded to a widespread call for the acknowledgement of complex interaction dynamics during conversation (*26*, *27*). We have demonstrated that multiscale similarity structure, as indexed by pink noise, explains variance in interlocutors’ enjoyment and sense of connection in conversations beyond that explainable with traditional convergence-based approaches and can predict real-world partner choice. We hope that this work will not just provide a new method for quantifying conversation but additionally serve as a springboard for future work in communications theory and practice that acknowledges the complex dynamic nature of conversation.

## MATERIALS AND METHODS

### Generating a conversation’s semantic similarity trajectory

All conversations analyzed in this paper first took place in the spoken modality and were then transcribed into text by various methods. For each conversation transcript, the text was split into turns, defined as any words contained in one speaker’s uninterrupted flow of speech (Fig. 1A; see Supplementary Text, “Transcription segmentation and embedding details” section for a discussion of the choice to segment on the level of the turn). Each turn was then passed through MPNet (*36*), a transformer model that analyzes how words relate to and influence each other within a sentence to capture its overall meaning. Specifically, we used *all-mpnet-base-v2*, a pretrained sentence-level version of the model hosted by Hugging Face (*37*) and instantiated with *SentenceTransformers* (*38*). MPNet converts each turn into a numerical representation: a vector of 768 numbers that defines that turn’s location within a high-dimensional semantic space where similar meanings cluster together (Fig. 1B, top). The cosine distance was then calculated between each turn’s embedding and that of the prior turn. For a conversation of length *n*, this produces a length *n* − 1 signal representing interlocutors’ movements toward and away from each other in semantic space (Fig. 1B, bottom). See Supplemental Text, “Transcription segmentation and embedding details” section, and fig. S2 for results from alternative embedding models.

### Identifying pink noise within semantic synchrony trajectories

We applied detrended fluctuation analysis (DFA) to each conversation’s semantic similarity trajectory to determine the signal’s noise scaling coefficient (*21*, *22*). Briefly, DFA analyzes how fluctuations in a signal scale across different timescales. It works by (i) creating a cumulative sum of the time series, (ii) dividing this into segments of length *n*, (iii) detrending each segment by subtracting its linear fit, (iv) calculating the root-mean-square fluctuation around zero for each segment length *n*, and (v) examining how these fluctuations scale with different values of *n*. The scaling relationship reveals whether the signal is dominated by short-range randomness or exhibits long-range temporal correlations. Scaling coefficients near 0.5 indicate white noise, coefficients near 1 represent pink noise, and coefficients near 1.5 represent red noise, or Brownian drift.

To control for systematic biases in scaling coefficients introduced by conversation length, we generated a conversation-specific null distribution of noise scaling coefficients by scrambling each conversation’s semantic similarity trajectory 1000 times. We then used this null distribution to compute a *z*-score (for each noise scaling coefficient, we subtracted the mean of its null distribution, then divided by the SD of the null distribution) for the original noise scaling coefficient, a measure which we call PNR (Fig. 1C).

### CANDOR corpus

The CANDOR corpus is a large, open dataset consisting of 1656 video-chat conversations in English between participants recruited online through Prolific (*5*). Data collection and experimental procedures were approved by Ethical & Independent Review Services, protocol no. 19160-0, and all participants provided informed consent both before and after taking part in the study. Participants were instructed to talk as if they had just met at a social event, for at least 25 min (mean length, 31 min; SD, 7.96 min). We used the Cliffhanger-generated transcripts provided with the corpus to calculate PNR scores for each conversation [(*5*); see Supplemental Text, “Transcription segmentation and embedding details” section for a discussion of this choice and results from an alternative transcription]. As an additional robustness check to confirm that the observed pink noise in these conversations’ semantic similarity trajectories is rooted in the original order of turns, not just temporally agnostic distributional statistics, we recalculated PNR after shuffling the order of turns in each conversation.

### Testing the dyadic nature of multiscale organization

First, we tested whether PNR was unique to the realized dyad or if it could be realized degenerately by generating interleaved pseudoconversations. First, we identified 1551 of the 1655 CANDOR conversations that could be matched with a different conversation by turn count. Then, we took the first speaker’s turns from the original conversation and replaced the second speaker’s replies with the second speaker’s turns from the length-matched conversation. Using this new pseudo-conversation transcript, we calculated PNR as before. To determine whether the interleaved conversations had systematically different PNRs from their original (or individually derived; described in the next paragraph) counterparts, we fit a linear model of form *PNR ∼ condition + (1|convo_idA) + (1|convo_idB)*, where *convo_idA* is the conversation ID for the original conversation and *convo_idB* is the conversation ID for the length-matched counterpart. For original conversations (i.e., noninterleaved ones), both conversation IDs in the random effects slots are the same. We additionally compared the mean and SD of semantic similarity values for each conversation to investigate the possible sources of high PNR values in interleaved conversations.

Second, we tested whether PNR values derived from an individual’s half of a conversation (i.e., just turns produced by a single speaker) would systematically differ from their dyadic counterparts. For each conversation, we generated an individual transcript for each participant, then calculated PNR as usual. We compared the PNRs for individual versus dyadic conversation transcripts with a linear model of the form *PNR* *∼ condition + (1|convo_id)*.

Third, we tested whether PNR behaved like a stable individual trait. To do this, we isolated the 94 participants who took part in five or more conversations (who participated in 1041 conversations total) and tested whether the dyadic PNR scores belonging to those conversations were more similar within-individual than across-individual by comparing the pairwise distances in PNRs to pairwise difference in identity using a Mantel test. In other words, we created two symmetric representational dissimilarity matrices, each with dimensions 1041 by 1041: one consisting of differences in PNR between each possible conversation pair (i.e., conversation A PNR minus conversation B PNR) and one consisting of a model of shared speaker identity (i.e., a distance of 0 if conversations A and B shared a speaker; a distance of 1 if they did not). We then vectorized the upper triangle of each matrix and Pearson correlated them. Last, we assessed significance nonparametrically by comparing the observed correlation coefficient to a null distribution generated by recomputing correlation after shuffling rows and columns of one of the dissimilarity matrices (*n* = 10,000 iterations).

Fourth, we tested whether individual and dyadic PNRs responded similarly to transcript-level disruptions targeting turn-handoff structure. For all dyadic and individual conversation transcripts, we recalculated PNR after permuting the location of turn breaks within the conversation. This transformation maintains the original order of words, number of turns, and distribution of turn lengths, disrupting only the coordinative give-and-take of the conversation. We then compared original PNRs to breakpoint-shuffled PNRs for dyadic and individual conversations using paired *t* tests and then compared the direction and magnitude of the *t* statistics.

Fifth, we compared the predictive weight of individual and dyadic PNR values in the prediction of enjoyment and connection. We conceptualized this in two ways. The first model compares the role of dyadic PNR, one’s own PNR, and one’s partner’s PNR on an outcome: *outcome ∼ PNR + PNR_self + PNR_other + (1|convo_id)*. The second recognizes that individual PNRs may affect outcomes in an individual-agnostic manner. More plainly, the average of the two speakers’ individual PNRs is still a dyad-level measure when it comes to predicting individual outcomes. We defined *average_individual_PNR* as *(PNR_self + PNR_other)/2*—leaving the only truly individual component as the delta between the individual PNR of the participant who generated the outcome rating and their partner’s—for a model of form *connection_self ∼ PNR + average_indiv_PNR + self_PNR_minus_other + (1|convo_id)*.

Just because individual PNR scores cannot outcompete dyadic PNR in a linear model does not necessarily mean that dyadic PNR could not be reduced to individual PNRs using a more robust, nonlinear equation. To test this, we constructed a symmetric model rooted in the mean individual PNR and the squared difference between the individual PNRs (supplementary Model 1.0.2). We then found the Pearson correlation between real dyadic PNRs and the dyadic PNRs predicted by this model.

Metastable systems are characterized by movement between distinct behavioral regimes, such that the system temporarily stabilizes in locally coherent states before transitioning to alternative states. Critically, it is not merely the presence of multiple regimes that defines metastability, but the structured temporal organization of transitions between them. To test whether the observed pink noise reflects this regime-switching architecture, we disrupted the temporal arrangement of high- and low-variance states while preserving their overall distribution. For each conversation’s semantic similarity trajectory, we calculated the variance of the signal over 10-turn sliding windows. We then defined two states of the system: one in which the variance is above median, another in which the variance is below median (fig. S4, A and B). (To be clear, we are not committed to conversation consisting solely of two states, high- and low-variance; we simply sought to test whether disrupting transitions between plausible states disrupts pink noise.) Were these regimes to represent genuinely different states of the system, we would expect to see differences in other statistical properties beyond variance. So, we tested whether regime-based chunks of semantic similarity trajectories exhibited a relationship between mean and variance with a model of the form *chunk_mean ∼ chunk_var + chunk_len + (1|convo_id)*. Then, to test whether these regime transitions affected pink noise, we disrupted them in two different ways. First, we split each state in half and interleaved them with their neighbors (fig. S4C). This preserved large chunks of the original semantic similarity signal while disrupting the order of state transitions. As an additional test, we sorted the signal by state such that all high- and low-variance chunks were next to each other (fig. S4D). We then calculated the PNRs of these disrupted signals and compared them to their intact counterparts with paired *t* tests.

We then checked that these variance-defined regimes were not merely inherited from individual speakers. We performed the same variance-based partitioning on the individual turn trajectories, aligned them to their dyadic counterparts, and calculated the Hamming similarity between an individual’s binary state trajectory and its dyadic counterpart. To generate a null distribution for each comparison, we recalculated Hamming similarity on an exhaustive circle shift. We then tested whether the *z*-scored Hamming similarities exceeded zero at a statistically significant level with a linear model of the form *overlap_z ∼ 1 + (1|convo_id)*. We included the random effect of conversation ID to mitigate potential violations of independence, given that each dyadic state trajectory is used twice. In case the median split was too coarse a measure to capture overlapping state structure between individual and dyadic variance trajectories, we additionally calculated Pearson correlation coefficients between each pair. Using the same circle shifted z-scoring technique just described, we tested whether the variance correlation *z*-scores significantly exceeded 0 with a model of the form *variance_correlation_z ∼ 1 + (1|convo_id)*. However, this model did not converge. After confirming that variance correlation *z*-scores were not correlated within conversation id, we dropped the random effect of conversation.

### Predicting conversation outcomes from PNR scores

Before and after each conversation, participants took extensive surveys about their personalities and experiences. We separated the 205 survey questions that resulted in numerical responses into six categories: those relating to (i) conversation enjoyment, (ii) sense of ongoing connection, (iii) engagement with and memory for the conversation, (iv) demographics and low-level conversation statistics (like the participant’s perception of the average turn length and which partner smiled more), (v) how each participant rated their partner on various trait batteries, and (vi) how each participant scored on those same trait scales themselves.

We ran two sets of analyses to identify relationships between a conversation’s PNR and the interlocutors’ survey results. First, we correlated a conversation’s PNR with each of the 205 numerical survey values (Pearson correlation for continuous variables, Spearman for Likert-scaled variables) which were averaged between the two speakers for a given conversation, meaning that each conversation contributed one datapoint to this analysis. We corrected for multiple comparisons in two ways: first with a conservative Bonferroni correction, then with the more lenient Benjamini/Hochberg FDR. After finding the sets of variables that survived the two different multiple hypothesis correction methods, we tested whether enjoyment- or connection-related variables were overrepresented in these sets using a *chi-square* test. In addition, we took the first principal component of the survey responses within each of our six question categories (replacing any missing values with the median value of the variable) and correlated them with PNR.

To test whether the relationship between PNR and outcome variables is robust to the inclusion of low-level conversation statistics, we calculated the following covariates: mean and SD of turn lengths (in seconds), mean and SD of turn word counts (in words), transcription quality proxy, and backchannel rate (only available for CANDOR). Transcription quality proxy was calculated by splitting each transcript into 100-word chunks, applying aiXplain’s *NoRefER* model (*39*) to each chunk, and averaging the automatic speech recognition (ASR) proxy scores across all chunks. For the CANDOR dataset, we calculated the backchannel rate by dividing the total number of backchannels (i.e., short verbal interjections such as “uh-huh” or “yeah”) as labeled by the Backbiter algorithm; (*5*) in a conversation divided by the total turn count. We then fit linear models to predict outcomes from a combination of PNR and basic controls (see Supplementary Models; Models 2.1.1 and 2.1.2).

We performed several additional control analyses to confirm that PNR could explain variance in conversation outcomes beyond that attributable to simpler features of the semantic similarity trajectory. For the enjoyment and connection categories, we modeled the first principal component of conversation-level outcome variables as a linear function of PNR and the mean, slope, and variance of a given conversation’s semantic similarity trajectory. The input variables were all *z*-scored using either scipy’s *zscore* function or the built-in R *scale* function to generate interpretable effect sizes. We additionally tested several more complex linear methods: ARMA models, dynamic time warping (DTW), and information theoretic coupling. Because these methods likely contained overlapping information and information that tiled the multiple scales that PNR assays, we tested each of them against PNR separately. For the ARMA modeling, we fit an ARMA (1,1) model to the semantic similarity trajectory of each conversation, saved the AR and MA coefficients, and then fit a linear model of the form *outcome ∼ PNR + AR + MA*. Next, we took each conversation, split the turns based on speaker, embedded each speaker’s turns into MPNet space, and calculated the DTW similarity score between the two embedding trajectories with *tslearn*’s DTW implementation (*40*) and fit a linear model of the form *outcome ∼ PNR + DTW*. Last, we calculated latent mutual information (LMI) between speakers’ turn embeddings using the *latentmi* Python package. We used LMI because the embedding dimensions were too high to calculate mutual information directly. To test whether PNR had explanatory power beyond mutual information, we fit a linear model of the form *outcome ∼ PNR + LMI*. We additionally tested whether PNR could explain unique variance beyond the aggregate family of these alternative linear methods with models of the form *outcome ∼ PNR + AR + MA + DTW + LMI*. To break down what scales of information were captured by these different linear alternatives, we additionally calculated the lag-1 autocorrelation function (ACF) and pitted it against ARMA and DTW separately: *outcome ∼ PNR + AR + MA + lag1_ACF* and *outcome ∼ PNR + DTW + lag1_ACF*.

### Interracial Friends corpus

The Interracial Friends corpus consists of 97 video-chat conversations between Black and non-Black friends recruited on college campuses who spoke for around 20 min (mean length, 18.0 min; SD, 9.3 min) about a formative experience that the Black friend shared at the beginning of the conversation. Data collection and experimental procedures were approved by Stanford IRB protocol no. 41090, and all participants provided informed consent before taking part in the study. All conversations were transcribed by professionals hired through Scribie. These participants also answered a battery of pre- and postconversation surveys from which we identified three sets of questions that captured conversational enjoyment, sense of connection, and individual participant traits. We correlated the first principal component of each of these categories with PNR across conversations.

### Speed dating corpus

The speed dating corpus consists of episodes from a speed-dating podcast called Blink Date (https://feeds.acast.com/public/shows/date-in-a-blink). After excluding conversations with fewer than 20 turns, the Blink Date corpus consists of 31 10-min-long audio-only phone conversations between two strangers. All but one of these conversations was transcribed by the podcast hosts, and the final transcript was produced using WhisperX (*41*) and diarized with NeMo (*42*). If at the end of the conversation both parties said “yes” or “maybe” to a second date, it was considered a match. We tested whether conversations that resulted in matches had higher average PNR values than those that did not result in matches with a one-tailed *t* test, followed by a one-sided linear mixed-effects model including conversation length in minutes and turn count.

### Longitudinal conversation corpus

The longitudinal conversation corpus consists of repeated conversations between a set of 25 dyads collected from podcasts. To be included in this corpus, a podcast had to meet a predetermined set of criteria regarding access, length, structure, quality, amateurism, and familiarity. Specifically, each podcast had to be accessible via a public RSS feed, consist of at least 50 episodes, have an average episode length of over 30 min, consist primarily of free-flowing conversations between two hosts (occasional guest episodes were acceptable, but formats in which hosts read text from social media posts or separated their conversations into strict segments were not), have no or minimal interruptions from advertisements, have positive reviews when available, be recorded by amateurs (which we defined as anyone who did not primarily make their living from podcasting, hosting radio, or acting at the time of starting their podcast) who do not otherwise speak to each other every day (e.g., married couples). After finding 25 podcasts that fit these criteria, we removed guest episodes, live episodes, and sponsored episodes (e.g., some podcasts allow paid subscribers to request specific topics). This left 1032 usable conversations which were then transcribed with WhisperX and diarized with NeMo (*42*). Last, we modeled conversation PNR as a linear function of episode number, with a random effect of podcast and a host of low-level covariates: duration in seconds, turn count, mean turn wordcount, SD of turn wordcounts, mean turn duration, SD of turn durations, and a proxy for automated transcription quality (ASR proxy).

### LLM generated conversations

We asked an LLM, Claude 3.7 Sonnet (*24*), to write 50 high- and 50 low-enjoyment dialogues between strangers who have just met at a coffee shop, each with 100 turns. These conversations were generated using the following prompt:

<system>Hi Claude! I’m running an experiment where I want to test the intuitions that you’ve developed by reading tons of human-generated text about what makes a conversation between two people feel like they’re building a connection and enjoying each other’s company — essentially what makes a conversation good. However, I don’t want you to explicitly tell me about your intuitions, I want you to write me some example good and bad conversations! Please write conversations that sound as natural and realistic as possible within the provided constraints. As a note, I’ve noticed that you have a tendency to mirror my writing style — please don’t do that here! Again, the purpose is to test the intuitions and representations that you’ve developed by reading lots of other people’s text. </system>

<instructions>

<item> I’m going to ask you to write 100 conversation scripts, 50 good conversations and 50 bad conversations.</item>

<item> Each conversation will take place between two strangers who have just met at a coffee shop.</item>

<item> These conversations should all be distinct from each other: do not repeat phrasing or structure, and the topics should be diverse. <thinking> Take a minute to think about 50 uniquely good conversations between strangers, and 50 uniquely bad conversations between strangers. Be creative!</thinking></item>

<item> Each conversation will consist of 100 turns, consisting of 50 back-and-forths labeled A1, B1, A2, B2 ... etc. until A50 and B50. </item>

<item> Do not add any additional speakers: these conversations are strictly between two people. </item>

<item> Do not use action tags describing what each person is doing. </item>

<item> Make sure that each turn contains at least one word of reply - no silences. </item>

<item> Save each of these conversation scripts as its own markdown artifact with a title that indicates condition (good or bad). When creating the markdown filenames, please add “no-specified-length” somewhere in the title.

</instructions>

<thinking> Please think about these and ask clarifying questions about anything you need! </thinking>

To confirm that third-party human readers agreed with Claude’s assessment of conversations’ enjoyability, we asked at least five online participants recruited with Prolific to read each conversation and rate how enjoyable the dialogue was on a scale of 1 to 5. Data collection and experimental procedures were approved by the Committee for the Protection of Human Subjects at Dartmouth College under Protocol no. 00032009, and all participants provided informed consent before taking part in the study. After confirming that human raters agreed with Claude’s assessment, we compared the PNRs of the high- and low-enjoyment conversations with an independent-samples *t* test.

## Supplementary Materials

### This PDF file includes:

Supplementary Text Figs. S1 to S7 Data Dictionary Supplementary Models: specifications and results References

## References

[1] E. E. Levine, A. R. Roberts, T. R. Cohen, Difficult conversations: Navigating the tension between honesty and benevolence. Curr. Opin. Psychol. 31, 38–43 (2020). 10.1016/j.copsyc.2019.07.034 31446340

[2] P. Dao, Effects of task goal orientation on learner engagement in task performance. IRAL Int. Rev. Appl. Linguist. Lang. Teach. 59, 315–334 (2021).

[3] C. N. Wright, Educational orientation and upward influence: An examination of students’ conversations about disappointing grades. Commun. Educ. 61, 271–289 (2012).

[4] E. M. Templeton, L. J. Chang, E. A. Reynolds, M. D. Cone LeBeaumont, T. Wheatley, Fast response times signal social connection in conversation. Proc. Natl. Acad. Sci. U.S.A. 119, e2116915119 (2022). 10.1073/pnas.2116915119 PMC8794835 35042815

[5] A. Reece, G. Cooney, P. Bull, C. Chung, B. Dawson, C. Fitzpatrick, T. Glazer, D. Knox, A. Liebscher, S. Marin, The CANDOR corpus: Insights from a large multimodal dataset of naturalistic conversation. Sci. Adv. 9, eadf3197 (2023). 10.1126/sciadv.adf3197 PMC10065445 37000886

[6] D. A. Coker, J. Burgoon, The nature of conversational involvement and nonverbal encoding patterns. Hum. Commun. Res. 13, 463–494 (1987).

[7] M. G. Frank, P. Ekman, W. V. Friesen, Behavioral markers and recognizability of the smile of enjoyment. J. Pers. Soc. Psychol. 64, 83–93 (1993). 10.1037//0022-3514.64.1.83 8421253

[8] A. S. Gabay, A. Pisauro, K. C. O’Nell, M. A. J. Apps, Social environment-based opportunity costs dictate when people leave social interactions. Commun. Psychol. 2, 42 (2024). 10.1038/s44271-024-00094-5 PMC11081926 38737130

[9] S. Tsang, K. Barrentine, S. Chadha, S. Oishi, A. Wood, Social exploration: How and why people seek new connections. Psychol. Rev. 132, 656–679 (2024). 10.1037/rev0000499 39264681

[10] C. Geishauser, C. van Niekerk, H. Lin, N. Lubis, M. Heck, S. Feng, M. Gašić, “Dynamic dialogue policy for continual reinforcement learning” in *Proceedings of the 29th International Conference on Computational Linguistics*, N. Calzolari, C.-R. Huang, H. Kim, J. Pustejovsky, L. Wanner, K.-S. Choi, P.-M. Ryu, H.-H. Chen, L. Donatelli, H. Ji, S. Kurohashi, P. Paggio, N. Xue, S. Kim, Y. Hahm, Z. He, T. K. Lee, E. Santus, F. Bond, S.-H. Na, Eds. (International Committee on Computational Linguistics, 2022), pp. 266–284; https://aclanthology.org/2022.coling-1.21.

[11] P. A. Heeman, “Representing the Reinforcement Learning state in a negotiation dialogue” in *2009 IEEE Workshop on Automatic Speech Recognition & Understanding* (2009), pp. 450–455; https://ieeexplore.ieee.org/document/5373413.

[12] M. Gallotti, C. D. Frith, Social cognition in the we-mode. Trends Cogn. Sci. 17, 160–165 (2013). 10.1016/j.tics.2013.02.002 23499335

[13] M. E. Ireland, R. B. Slatcher, P. W. Eastwick, L. E. Scissors, E. J. Finkel, J. W. Pennebaker, Language style matching predicts relationship initiation and stability. Psychol. Sci. 22, 39–44 (2011). 10.1177/0956797610392928 21149854

[14] A. L. Gonzales, J. T. Hancock, J. W. Pennebaker, Language style matching as a predictor of social dynamics in small groups. Commun. Res. 37, 3–19 (2010).

[15] C.-W. Yu, Y.-S. Chuang, A. N. Lotsos, T. Meier, C. M. Haase, The more similar, the better? Associations between latent semantic similarity and emotional experiences differ across conversation contexts. J. Lang. Soc. Psychol. 44, 961–984 (2025).

[16] P. J. Taylor, S. Thomas, Linguistic style matching and negotiation outcome. Negot. Confl. Manag. Res. 1, 263–281 (2008).

[17] R. Dale, M. J. Spivey, Unraveling the Dyad: Using recurrence analysis to explore patterns of syntactic coordination between children and caregivers in conversation. Lang. Learn. 56, 391–430 (2006).

[18] R. Fusaroli, K. Tylén, Investigating conversational dynamics: Interactive alignment, interpersonal synergy, and collective task performance. Cognit. Sci. 40, 145–171 (2016). 10.1111/cogs.12251 25988263

[19] D. H. Abney, A. Paxton, R. Dale, C. T. Kello, Complexity matching in dyadic conversation. J. Exp. Psychol. Gen. 143, 2304–2315 (2014). 10.1037/xge0000021 25285431

[20] M. T. Tolston, M. A. Riley, V. Mancuso, V. Finomore, G. J. Funke, Beyond frequency counts: Novel conceptual recurrence analysis metrics to index semantic coordination in team communications. Behav. Res. Methods 51, 342–360 (2019). 10.3758/s13428-018-1132-6 30284212

[21] C.-K. Peng, S. V. Buldyrev, S. Havlin, M. Simons, H. E. Stanley, A. L. Goldberger, Mosaic organization of DNA nucleotides. Phys. Rev. E 49, 1685–1689 (1994). 10.1103/physreve.49.1685 9961383

[22] L. Rydin Gorjão, G. Hassan, J. Kurths, D. Witthaut, MFDFA: Efficient multifractal detrended fluctuation analysis in python. Comput. Phys. Commun. 273, 108254 (2022).

[23] G. C. Van Orden, H. Kloos, S. Wallot, “Living in the pink: Intentionality, wellbeing, and complexity” in *Philosophy of Complex Systems*, C. Hooker, Ed. (Elsevier, 2011), pp. 629–672; www.sciencedirect.com/science/article/pii/B9780444520760500225.

[24] Anthropic, Claude 3.7 Sonnet; www.anthropic.com/claude/sonnet.

[25] D. Angus, Recurrence methods for communication data, reflecting on 20 years of progress. Front. Appl. Math. Stat. 5, 54 (2019).

[26] U. Hasson, C. D. Frith, Mirroring and beyond: Coupled dynamics as a generalized framework for modelling social interactions. Philos. Trans. R. Soc. Lond. B Biol. Sci. 371, 20150366 (2016). 10.1098/rstb.2015.0366 PMC4843605 27069044

[27] T. Wheatley, M. A. Thornton, A. Stolk, L. J. Chang, The emerging science of interacting minds. Perspect. Psychol. Sci. 19, 355–373 (2024). 10.1177/17456916231200177 PMC10932833 38096443

[28] R. Fusaroli, J. Rączaszek-Leonardi, K. Tylén, Dialog as interpersonal synergy. New Ideas Psychol. 32, 147–157 (2014).

[29] E. Tognoli, M. Zhang, A. Fuchs, C. Beetle, J. A. S. Kelso, Coordination dynamics: A foundation for understanding social behavior. Front. Hum. Neurosci. 14, 317 (2020). 10.3389/fnhum.2020.00317 PMC7457017 32922277

[30] I. Ravreby, Y. Shilat, Y. Yeshurun, Liking as a balance between synchronization, complexity and novelty. Sci. Rep. 12, 3181 (2022). 10.1038/s41598-022-06610-z PMC8873358 35210459

[31] J. Michalsky, H. Schoormann, “Pitch convergence as an effect of perceived attractiveness and likability” (2017), pp. 2253–2256; www.isca-archive.org/interspeech_2017/michalsky17_interspeech.html.

[32] J. L. Lakin, T. L. Chartrand, Using nonconscious behavioral mimicry to create affiliation and rapport. Psychol. Sci. 14, 334–339 (2003). 10.1111/1467-9280.14481 12807406

[33] S. E. Brennan, H. H. Clark, Conceptual pacts and lexical choice in conversation. J. Exp. Psychol. Learn. Mem. Cogn. 22, 1482–1493 (1996). 10.1037//0278-7393.22.6.1482 8921603

[34] S. E. Brennan, Lexical entrainment in spontaneous dialog (1996).

[35] C. Welker, T. Wheatley, G. Cason, C. Gorman, M. Meyer, Self-views converge during enjoyable conversations. Proc. Natl. Acad. Sci. U.S.A. 121, e2321652121 (2024). 10.1073/pnas.2321652121 PMC11513911 39401349

[36] K. Song, X. Tan, T. Qin, J. Lu, T.-Y. Liu, MPNet: Masked and permuted pre-training for language understanding. arXiv:2004.09297 [cs.CL] (2020).

[37] HuggingFace, all-mpnet-base-v2 Model Card (2024); https://huggingface.co/sentence-transformers/all-mpnet-base-v2.

[38] N. Reimers, I. Gurevych, Sentence-BERT: Sentence embeddings using Siamese BERT-Networks. arXiv:1908.10084 [cs.CL] (2019).

[39] K. A. Yuksel, T. Ferreira, A. Gunduz, M. Al-Badrashiny, G. Javadi, “A reference-less quality metric for automatic speech recognition via contrastive-learning of a multi-language model with self-supervision” in *2023 IEEE International Conference on Acoustics, Speech, and Signal Processing Workshops (ICASSPW)* (IEEE, 2023), pp. 1–5; https://ieeexplore.ieee.org/document/10193003.

[40] R. Tavenard, J. Faouzi, G. Vandewiele, F. Divo, G. Androz, C. Holtz, M. Payne, R. Yurchak, M. Rußwurm, K. Kolar, E. Woods, Tslearn, a machine learning toolkit for time series data. J. Mach. Learn. Res. 21, 1–6 (2020).

[41] M. Bain, J. Huh, T. Han, A. Zisserman, “WhisperX: Time-accurate speech transcription of long-form audio” (2023), pp. 4489–4493; www.isca-archive.org/interspeech_2023/bain23_interspeech.html.

[42] M. Ashraf, Whisper Diarization: Speaker Diarization Using OpenAI Whisper (2024); https://github.com/MahmoudAshraf97/whisper-diarization.

[43] R. Fusaroli, K. Tylén, K. Garly, J. Steensig, M. H. Christiansen, M. Dingemanse, “Measures and mechanisms of common ground: Backchannels, conversational repair, and interactive alignment in free and task-oriented social interactions” in *Proceedings of the 39th Annual Conference of the Cognitive Science Society* (Cognitive Science Society, 2017), pp. 2055–2060.

[44] M. Dingemanse, N. J. Enfield, Interactive repair and the foundations of language. Trends Cogn. Sci. 28, 30–42 (2024). 10.1016/j.tics.2023.09.003 37852803

[45] C. A. Bergey, S. DeDeo, From “um” to “yeah”: Producing, predicting, and regulating information flow in human conversation. arXiv:2403.08890 [cs.CL] (2024).

[46] J. Ni, C. Qu, J. Lu, Z. Dai, G. H. Ábrego, J. Ma, V. Y. Zhao, Y. Luan, K. B. Hall, M.-W. Chang, Y. Yang, Large dual encoders are generalizable retrievers. arXiv:2112.07899 [cs.IR] (2021).

[47] L. Wang, N. Yang, X. Huang, B. Jiao, L. Yang, D. Jiang, R. Majumder, F. Wei, Text embeddings by weakly-supervised contrastive pre-training. arXiv:2212.03533 [cs.CL] (2024).

[48] T. Formal, C. Lassance, B. Piwowarski, S. Clcinchant, From distillation to hard negative sampling: Making sparse neural IR models more effective. arXiv:2205.04733 [cs.IR] (2022).

[49] T. Mikolov, K. Chen, G. Corrado, J. Dean, Efficient estimation of word representations in vector space. arXiv:1301.3781 [cs.CL] (2013).

[50] B. M. Goldstein, S. M. Burns, F. C. Peck, R. Dale, M. D. Lieberman, Cross-recurrence quantification analysis captures inter-brain coupling during naturalistic negotiation: A new dynamic approach for hyperscanning. Front. Neurosci. 19, 1713357 (2025). 10.3389/fnins.2025.1713357 PMC12833299 41601530

