# Supplementary material for: Pink noise in dyads’ semantic similarity trajectories predicts conversation enjoyment
Source: Sci Adv. 2026 Nov 6;12(45):eaeb2684. doi: 10.1126/sciadv.aeb2684 (PMC13742169; doi:10.1126/sciadv.aeb2684)
Supplement: Supplementary file 1 [file sciadv.aeb2684_sm.pdf]

Science Advances
AAAS

Supplementary Materials for

Pink noise in dyads' semantic similarity trajectories predicts conversation enjoyment

Kathryn C. O'Nell *et al.*

Corresponding author: Kathryn C. O'Nell, kathryn.coburn.onell@gmail.com;
Emily S. Finn, emily.s.finn@dartmouth.edu

Sci. Adv. **12**, eaeb2684 (2026)
DOI: 10.1126/sciadv.aeb2684

This PDF file includes:

Supplementary Text
Figs. S1 to S7
Data Dictionary
Supplementary Models: specifications and results
References

Supplementary Text

Transcription segmentation and embedding details

While there are many potential (and valid) ways to segment transcripts for embedding, we choose to embed on the level of the turn. This is because we see turns as the most meaningful level at which coordination occurs: when one person verbally responds to their partner. Were we to embed on the level of sentence, we would be additionally capturing how individuals internally coordinate their speech acts within a single turn. And were we to take a sliding window approach, we would lose important speaker-label information. To be clear, there are very likely interesting scale-free dynamics occurring at these different levels of analysis, but we hold that embedding at the level of the turn best represents interactive dynamics.

In the same vein, we use a version of the CANDOR transcripts, Cliffhanger, that treats backchannels as their own turns. There is a growing body of literature suggesting that backchannels serve an important role in regulating coordinative structure in conversation (*43*–*45*), and we thought it was worth including that information where possible. However, it is possible that backchannels are simply introducing short turns, creating pink noise artifacts. To check this possibility, we reran our analyses on the CANDOR dataset using Backbiter transcripts, which separate backchannels. Not only did we still recover pink noise (mean PNR = 1.1, SD = 1.2), but the PNRs still predicted conversation enjoyment (Pearson $r = 0.09$, $p = 0.001$).

We also sought to confirm that results were robust to the choice of embedding model. For our main analyses, we use all-mpnet-base-v2, but also repeated our analyses of the CANDOR dataset using three different transformer models -- GTR-T5-base (*46*), E5-large-v2 (*47*) , splade-cocondenser-ensembledistil (*48*) -- and a simple bag of words model (Word2Vec; *(49)*). For all four transformer models, but not Word2Vec, the first PC of participants' enjoyment and connection ratings correlated significantly with PNR of the semantic similarity trajectory derived from the transcript embeddings (Fig S2). This suggests that outcome-relevant semantic similarity trajectories depend on more than just word meaning, but on the context-aware embeddings of a whole turn.

Testing whether disrupting interactive properties of conversation reduces pink noise

As we consider pink noise to be a candidate metric of coordinative health, we conducted three robustness checks intended to demonstrate that disrupting the interactive and metastable aspects of conversation also reduces pink noise. First, we shuffled the order of turns in each conversation. We did this by separating each conversation into utterance-based turns, removing speaker labels, randomizing the order of the conversation, and adding the original speaker labels to the conversation. This maintains the total number of turns, distribution of turn lengths, and exact wording of each turn – it just disrupts the coordinative order. We did this once for each conversation, then calculated a pink noise robustness score for the disrupted conversation and compared this to its original counterpart with a paired t-test ($t=15.2$, $p <0.001$; Fig S3, left). However, we also wanted to test whether the overall arc of the conversation, regardless of where turn breaks occurred, might also produce comparable pink noise. For this analysis, we combined all turns into a speaker-agnostic transcript. We then made a list of the word counts of each turn in

the original conversation, shuffled those, and re-applied them to the transcript to generate disrupted turns. This maintains the total number of turns, distribution of turn lengths, and original order of words in the conversation. As in the previous analysis, we conducted this disruption once for each conversation and compared the new conversation's PNR to its original counterpart ($t=12.0$, $p<0.001$; Fig S3ß, right).

Testing the non-linearity of semantic similarity trajectories

While pink noise is defined in terms of a signal's linear autocorrelation structure, the presence of non-linearity in a conversation's semantic similarity trajectory provides evidence in favor of non-linear generating processes (i.e., non-linear dynamics between the two speakers). We tested for nonlinearity in two ways. First, we calculated each conversation trajectory's Brock-Dechert-Sheinkman (BDS) statistic. However, we wanted to make sure that any evidence of nonlinearity we saw in these results was not an artifact of the power spectrum, so we generated 1,000 IAAFT surrogates for each conversation's semantic similarity trajectory – signals with the same power spectrum, but without nonlinear behavior. We then compared the original signal's B-statistic against those of the IAAFT surrogates, generating a z-scored B-statistic for each conversation. We then performed Stouffer's combination test on the distribution of z-scored B-statistics, which showed evidence of nonlinearity ($p<0.001$). For our second non-linearity check, we tested whether semantic similarity trajectories could be modelled linearly, fitting an ARMA(1,1) model for each trajectory. These surrogates largely captured the linear autocorrelation function of the original signals, passing Ljung-Box tests (99.7% of conversations failed to reject the null). However, they failed to capture the non-Gaussian structure of the signals, failing Jarque-Bera tests (only 0.06% failed to reject the null). This indicates the presence of higher-order structure not explained by a linear model, consistent with nonlinear dynamics.

While not strictly a test of linearity, we also tested whether the conversation similarity trajectories exhibited state-dependent heteroskedasticity, which is consistent with multiplicative, interaction-dominant dynamics *(31).* This analysis was motivated by our earlier segmentation of conversations into variance-defined blocks (see previous section). Because metastable systems exhibit regime-dependent variability, we divided each similarity trajectory into high- and low-variance segments using a median split and calculated the average duration of each regime. Although this threshold is arbitrary, metastability predicts that variance-defined regimes should differ systematically in other statistical properties. For each variance-defined block, we calculated the block's variance and mean similarity (see Fig S6) and fit a linear model of the form mean_similarity ~ variance + chunk_length + (1|conversation_id). Consistent with our prediction, variance was significantly predictive of mean (coef = 2.04, $p < 0.001$) independent of the number of turns the conversation spent in a given state (coef = -0.0009, $p<0.001$). Additionally, we tested whether this positive relationship could be explained by the bounded nature of the mean cosine signal between 0 and 1. If the relationship was a confound of boundedness, we would expect to see many datapoints at the maximum variance level for each mean cosine distance (i.e. mean*(1-mean) via Popoviciu's inequality). However, the data did not approach this limit.

Comparison to other state-of the-art metrics of non-linear interaction

We additionally sought to compare the outcome-relevant information captured by PNR to that captured by other state-of-the-art measures of non-linear interaction: specifically, cross-recurrence quantification analysis (CRQA) (*50*). We performed this analysis in two ways. First, we computed multivariate CRQA on each conversation: we embedded each participant's turns in MPNet space, computed a pairwise cosine similarity matrix, set the recurrence threshold such that 3.5% of cross-speaker pairs were marked recurrent, restricted recurrence to a 10-turn lag window, and computed percentage recurrence and determinism. In the original formulation of CRQA, recurrent and determinism were (as expected) significant predictors of enjoyment, and determinism was a predictor of connection – but PNR still contributed significant independent explanatory power in both cases. (In a linear model of form enjoyment ~ PNR + recurrence + determinism, PNR: coeff=0.27, $p=0.002$; recurrence: coeff=-0.28, $p=0.001$; determinism: coef=0.61, $p<0.001$. In a linear model of form connection ~ PNR + recurrence + determinism, PNR: coeff=0.18, $p=0.007$; recurrence: coeff=0.02, $p=0.69$; determinism: coef=0.37, $p<0.001$)

We then repeated the analysis using continuous analogues to avoid thresholding. Recurrence was defined as the mean cosine similarity across all cross-speaker turn pairs. Determinism was defined by evaluating whether each cell's similarity was locally sustained along the forward diagonal: a cell received support from a diagonal neighbor equal to the minimum of the two similarity values, ensuring that support was bounded by the weaker value. If a cell had support from both diagonal directions, we took the maximum support (analogous to requiring at least one matching diagonal neighbor). Diagonal support was summed across the matrix and normalized by total recurrence. In the continuous formulation of CRQA, recurrence and determinism were (as expected) significant predictors of enjoyment and connection – but PNR still contributed significant independent explanatory power in both models. (In a linear model of form enjoyment ~ PNR + recurrence + determinism, PNR: coeff=0.25, $p=0.005$; recurrence: coeff=-0.51, $p<0.001$; determinism: coef=0.27, $p=0.003$. In a linear model of form connection ~ PNR + recurrence + determinism, PNR: coeff=0.14, $p=0.003$; recurrence: coeff=-0.46, $p<0.001$; determinism: coef=0.20, $p=0.007$). Together, these results indicate that PNR provides additional information about the aspects of nonlinear coupling structure of conversation that promote enjoyment beyond those encoded in recurrence and determinism.

Supplementary Figures

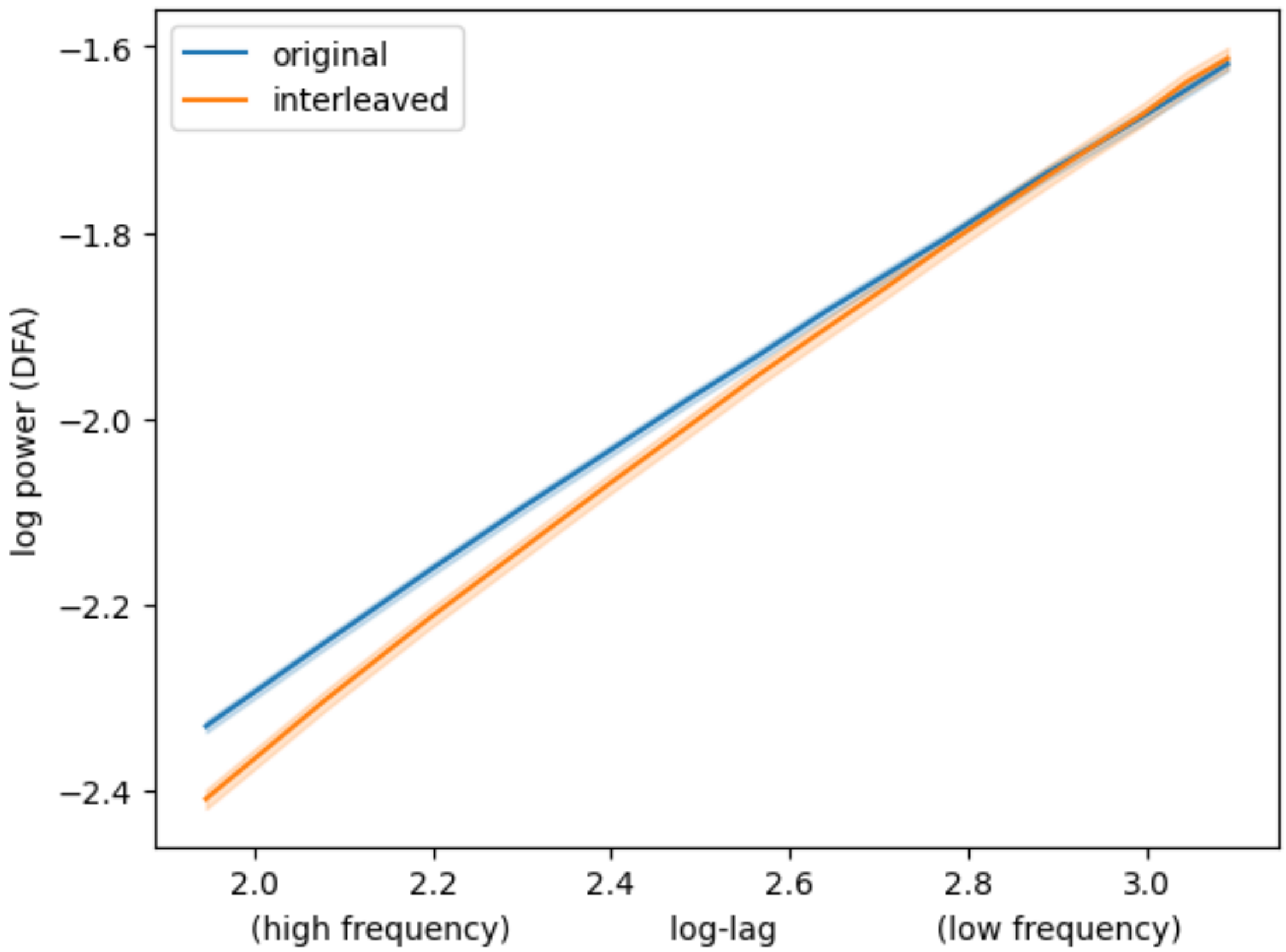

Fig S1. Frequency vs power distributions for interleaved vs original conversations

Pseudo-conversations consisting of interleaved turns from two separate conversations tend to exhibit higher pink noise robustness (PNR) scores than their original counterparts. When we look at their respective relationships between frequency and power in aggregate, we see that

interleaved conversations do not differ in their low frequency content, but are instead pushed towards lower power for high frequency fluctuations.

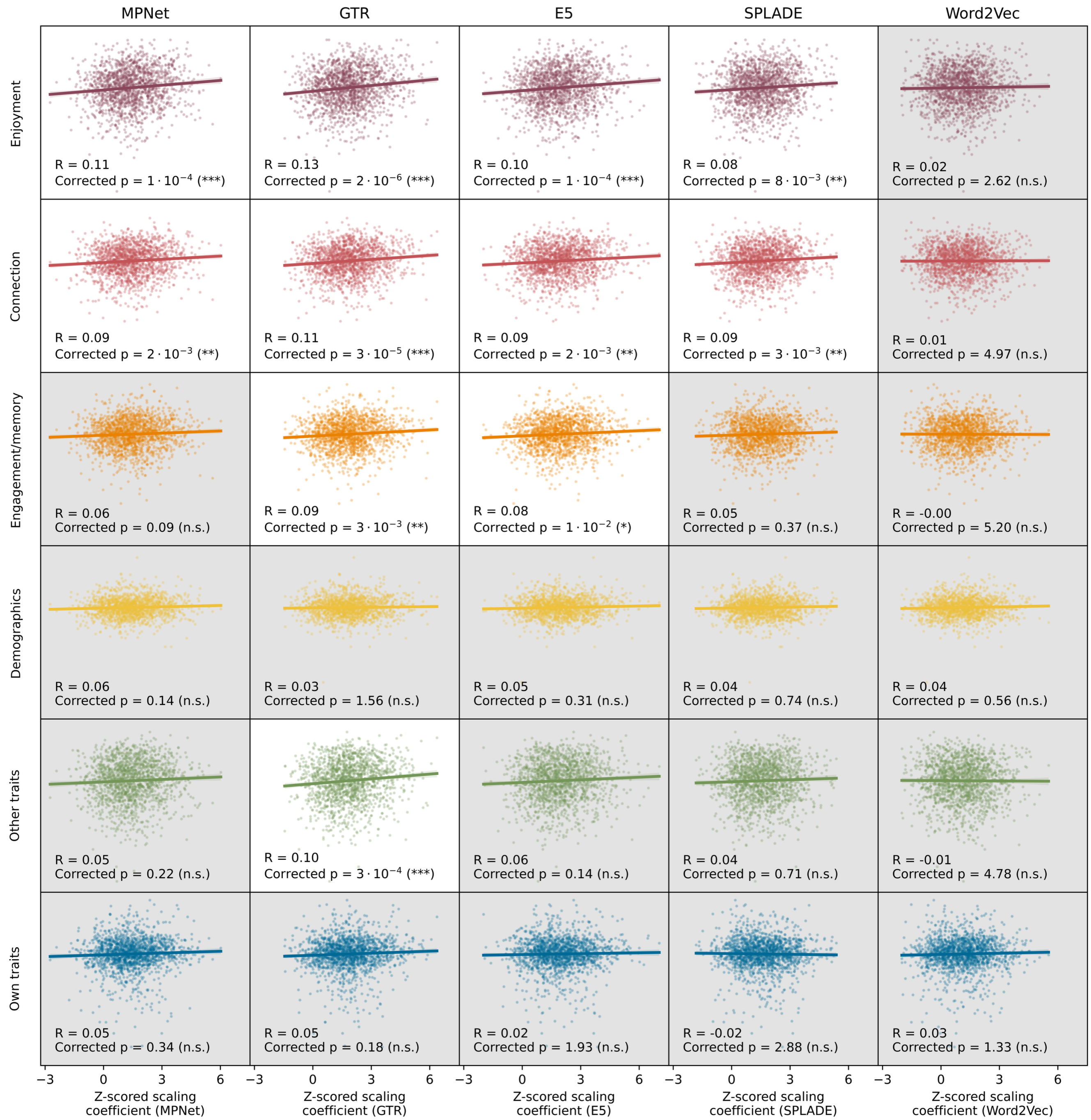

Fig. S2. Correlations between CANDOR conversation outcomes and pink noise robustness scores from five different embedding models. To test the robustness of our results to the choice of embedding model, we compared our original results (MPNet) to those generated by embedding conversations with three other transformer models (GTR, E5, SPLADE) and one bag of words model (Word2Vec). For all four transformer models, but not Word2Vec, the first PC of participants' enjoyment and connection ratings correlated significantly with PNR of the semantic similarity trajectory derived from the transcript embeddings. Statistics are reported after

Bonferroni correction, as PNR was tested against each of the 5 outcome variables without *a priori* hypotheses. Significance per Pearson correlation is indicated as following: * p < 0.05, ** p < 0.01, *** p < 0.001.

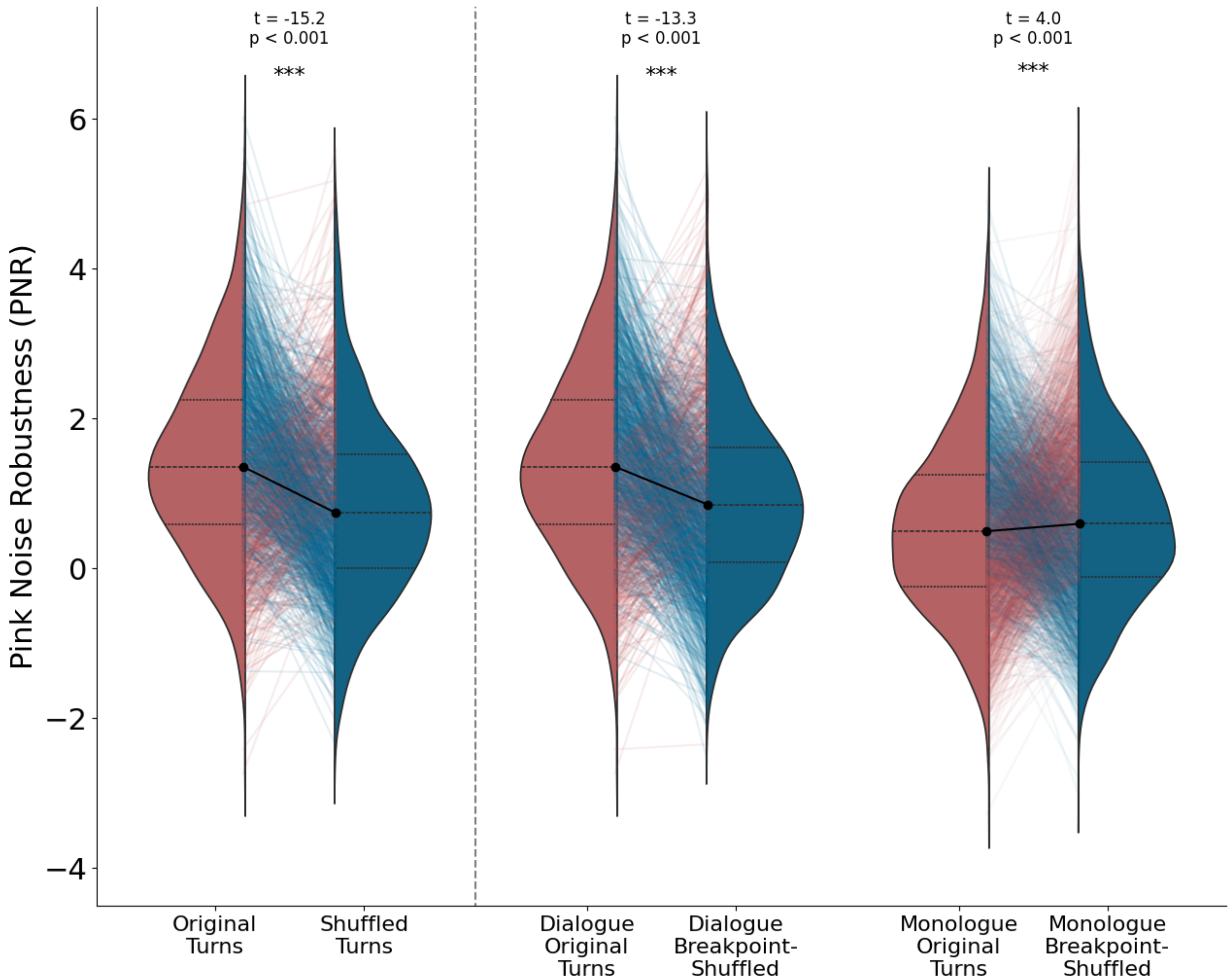

Fig. S3. Pairwise comparisons between PNRs of original CANDOR conversations versus conversations with (left) turn order shuffled, (middle) breakpoint locations shuffled, and breakpoint shuffles on monologues (right). (**Left**) First, we shuffled the order of turns in each conversation by separating the conversation into utterance-based turns, removing speaker labels, randomizing the order of turns, and adding the original speaker labels to the conversation. This maintains the total number of turns, distribution of turn lengths, and exact wording of each turn – it just disrupts the coordinative order. We did this once for each conversation, then calculated a pink noise robustness score for the disrupted conversation and compared this to its original counterpart with a paired t-test (t=-15.2, p <0.001). (**Middle**) For this analysis, we combined all turns into a speaker-agnostic transcript. We then made a list of the word counts of each turn in the original conversation, shuffled those, and re-applied them to the transcript to generate disrupted turns. This maintains the total number of turns, distribution of turn lengths, and original order of words in the conversation. As in the previous analysis, we conducted this disruption once for each conversation and compared the new conversation's PNR to its original counterpart (t=-12.0, p<0.001). (**Right**) For this analysis, we repeated the procedure for the

dyadic breakpoint shuffling, except on a single speaker's turns. In this case, the PNR was not significantly reduced, but in fact slightly increased ($t=4.0$, $p<0.001$). Significance per paired t-test is indicated as following: * $p < 0.05$, ** $p < 0.01$, *** $p < 0.001$.

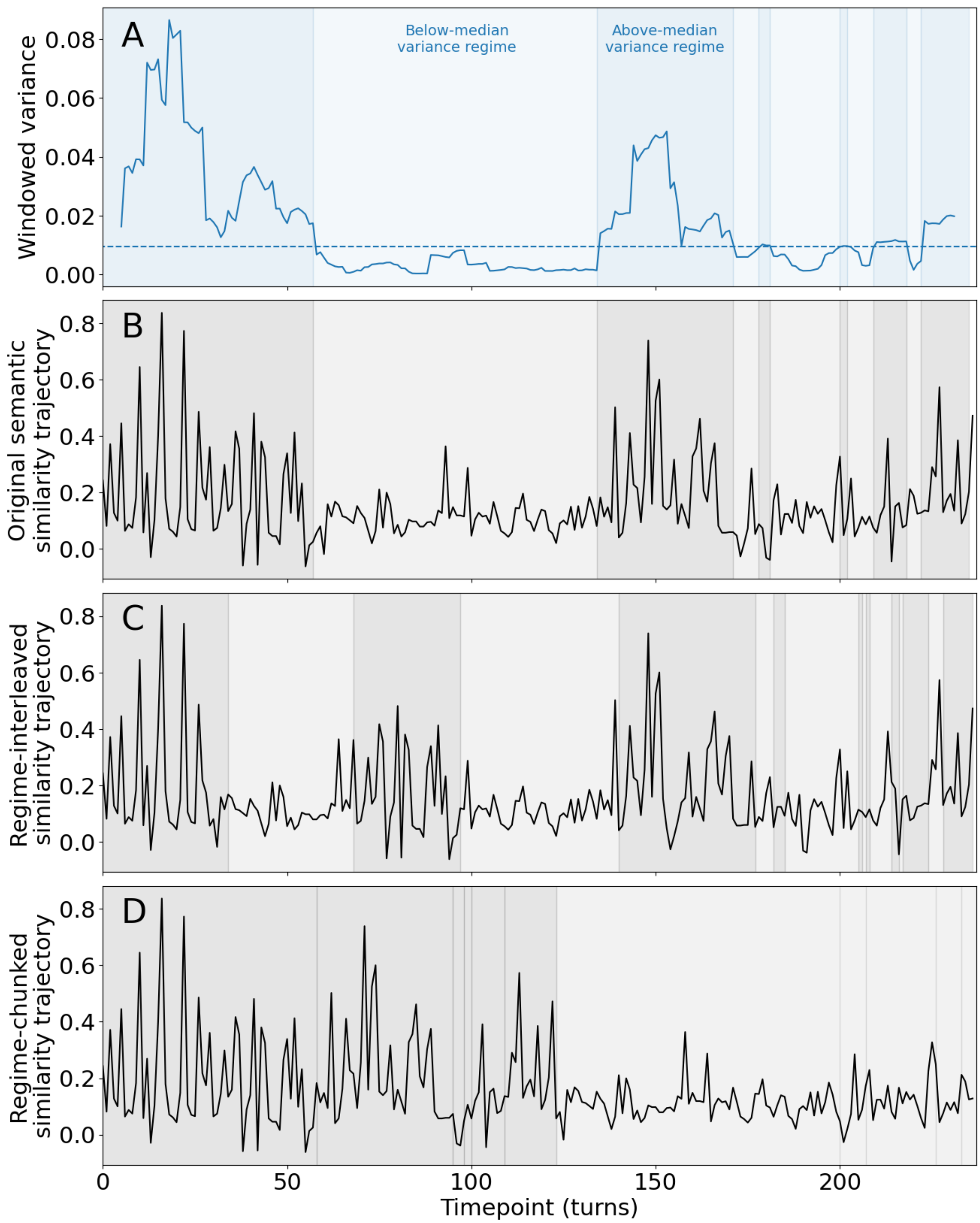

Fig S4. Variance-derived state-based shuffling paradigm For each CANDOR conversation's semantic similarity trajectory, we calculated the variance of the signal over 10-turn sliding windows. We then defined two states of the system: one in which the variance is above-median, another in which the variance is below-median. An example conversation is shown with its

windowed variance in (**A**) and original semantic similarity trajectory in (**B**). We then disrupted these transitions in two different ways. (**C**) First, we split each state in half and interleaved them with their neighbors This preserved large chunks of the original semantic similarity signal while disrupting the order of state transitions. (**D**) As an additional test, we sorted the signal by state such that all high- and low-variance chunks were next to each other.

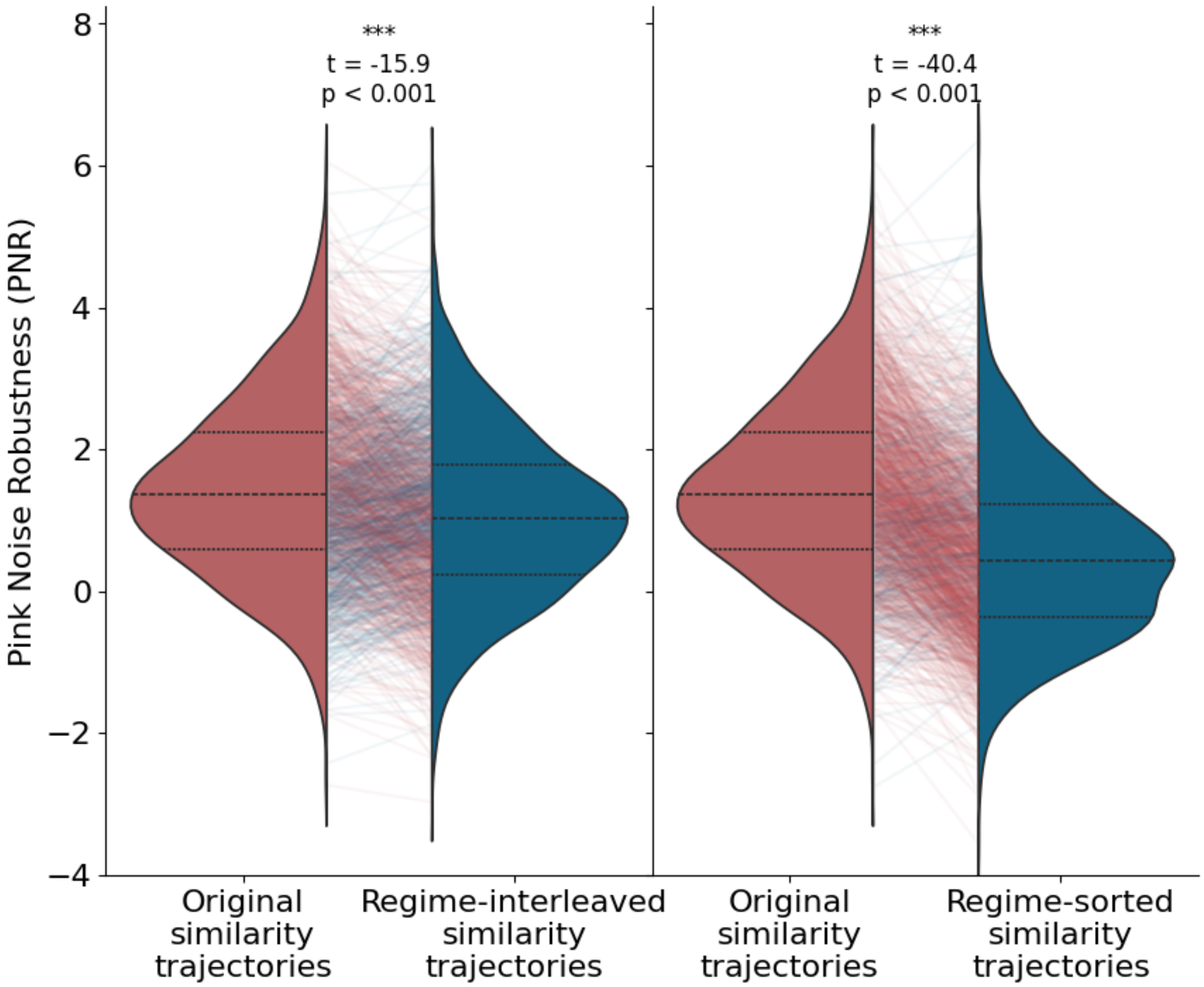

Fig. S5. Pairwise comparisons between PNRs of original conversations, and conversations with variance-derived states (left) interleaved, and (right) sorted. In this analysis, we calculated a 10-turn windowed variance trajectory for each CANDOR conversation's semantic similarity timeseries, then split the signal into two regimes: above-median variance, and below-median variance. We then disrupted each chunk of each regime in two ways (**left**) interleaving, and (**right**) sorting (see Fig. S4), and calculated PNRs for the disrupted semantic similarity trajectories. In both conditions, these disrupted PNRs are systematically lower than their original counterparts when compared with a paired t-test. Significance is indicated as following: * $p < 0.05$, ** $p < 0.01$, *** $p < 0.001$.

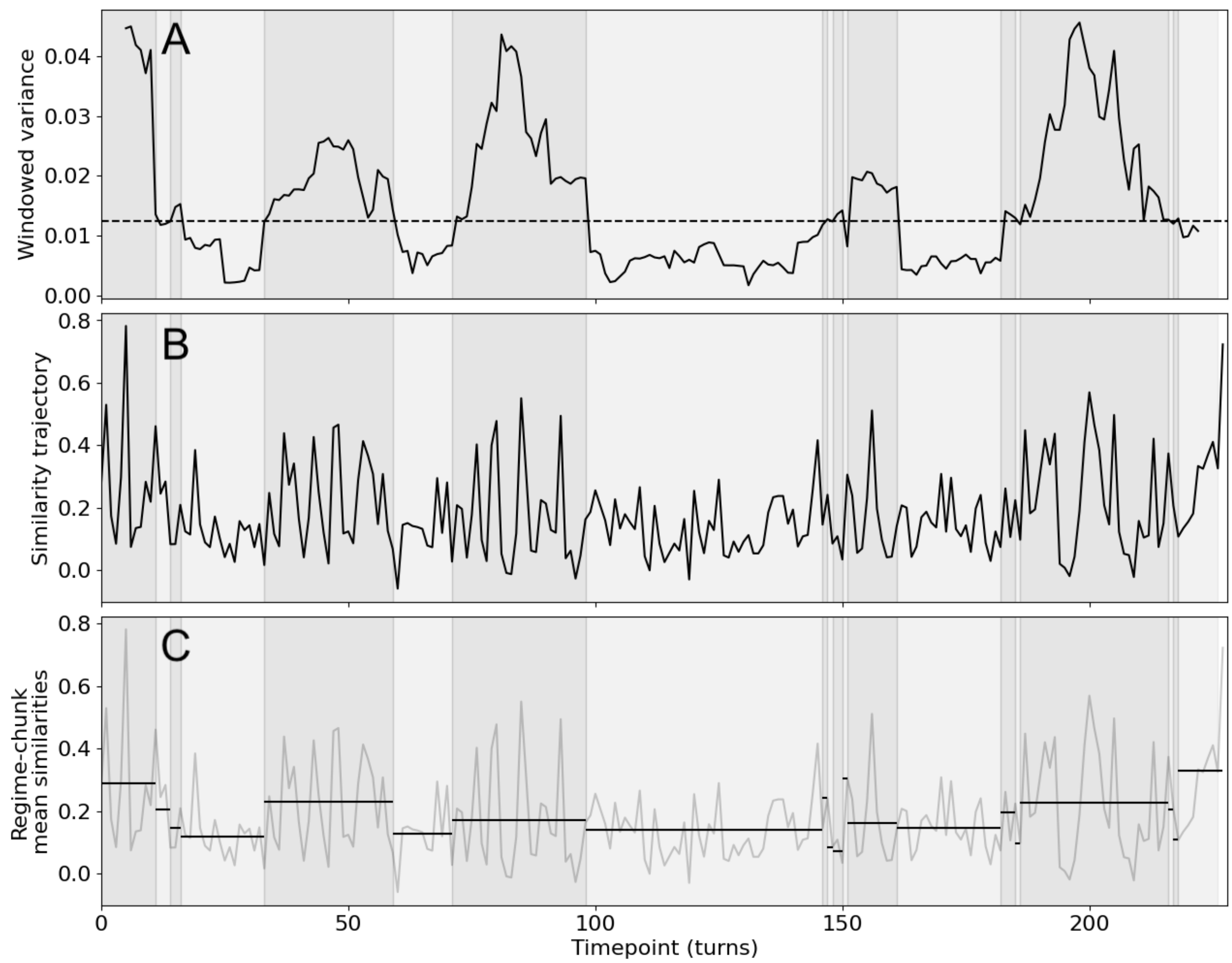

Fig. S6. Identifying systematic differences in mean similarity between high- and low-variance regimes of semantic similarity signal (**A**) In order to separate the semantic similarity signal into states, we generate a 10-turn sliding window measure of variance, then split along the median. (**B**) Then, we apply these state boundaries to the original similarity trajectory signal. (**C**)

For each state, we calculate mean similarity in order to test whether high-variance states differ in mean.

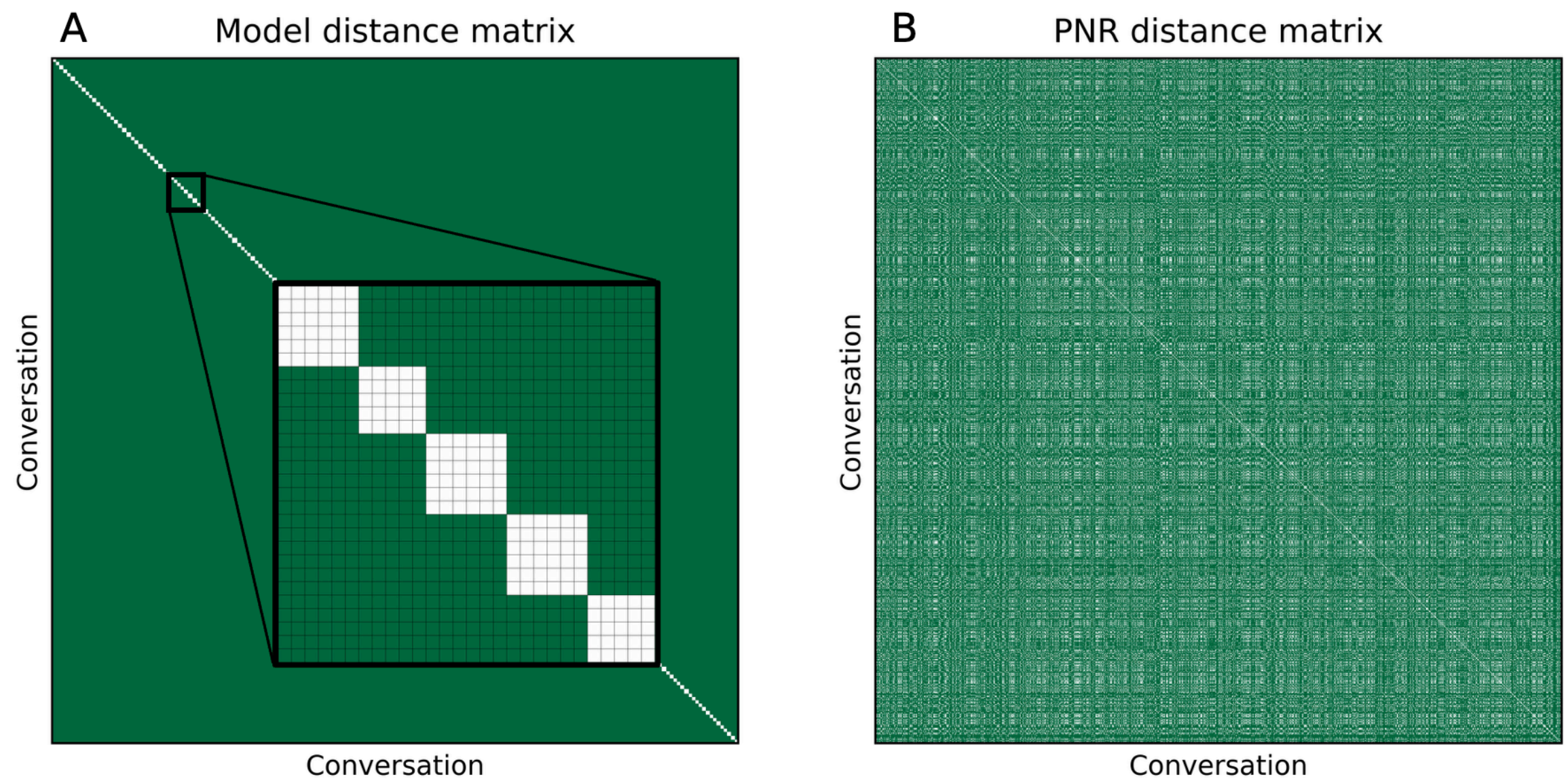

Fig. S7. Model vs real distance matrices between PNRs for the same participants across multiple conversations. For this analysis, we took the subset of CANDOR participants who took part in at least 5 conversations (*N*=94). We then compared two matrices: (**A**) the model distance matrix, in which participant has a distance of 0 from other conversations they participated in and a distance of 1 from any conversation they were not part of, and (**B**) the actual pairwise difference (absolute value) in PNR between conversations. These two matrices were used to test whether PNR scores were more similar within-individual than across-individual (p=0.25).

Data Dictionary

All data and code required to replicate the figures and statistics in this paper can be found at https://osf.io/7ju2s/ (DOI 10.17605/OSF.IO/7JU2S; data), and https://github.com/KatieONell/pink-noise (code). This includes two code notebooks: figures-and-statistics.ipynb which re-generates all data figures and statistics, and quickstart-and-tutorial.ipynb which is a tutorial for how to generate the noise scaling coefficient for a conversation transcript. Additionally, there are five folders containing the data for each section of the paper.

<u>candor-and-interracial-friends</u> contains:

- *arma-data.json*: contains AR and MA estimates for the semantic similarity trajectories of each conversation, along with associated statistics
- *candor-dwell-chunking-pnrs.json*: contains noise scaling coefficients and PNR values for similarity trajectories reorganized (sorted or interleaved) by variance-defined chunks
- *candor-PCs.json*: for each conversation ID, includes noise scaling coefficient (Hurst coefficient), pink noise robustness score (PNR), the conversation's similarity trajectory, all covariates, values for the first principal component of the six survey categories

(Enjoyment, Connection, Engagement/memory, Own traits, Other traits, Demographics/low-level) averaged across both participants, and a sub-dictionary containing PNR and survey PC values for individual participants in the conversation, keyed by their individual participant IDs

- *candor-shuffled-turns.json*: contains noise scaling coefficients, PNR values, similarity trajectories, and the new order of turns for turn-shuffled surrogate conversations
- *candor-survey-correlations.csv*: for each of the 205 survey variables in the Candor dataset, this file contains its category assignment, the number of individual responses, the number of conversations with at least one response, the number of unique answers (for determining whether to use Pearson or Spearman correlation), which correlation metric we used, the absolute value of the correlation coefficient, the p value of the correlation, which direction the correlation is in, and whether this correlation passes FDR and Bonferroni correction respectively
- *convo2dwell.json*: for each conversation, the similarity trajectory, size of bins used the create variance-defined regimes, the variance trajectory, the state at each turn, and the length of time spent in each regime before switching
- *dialogue-breakpoint-shuffle.json*: for each breakpoint-shuffled conversation, the updated similarity trajectory, noise scaling coefficient, PNR value – and the original PNR value to compare against
- *dyadic-regime-chunk-data.csv*: for each variance-defined chunk across all conversations, the chunk mean similarity, chunk variance, the length (in turns) of the chunk, and the conversation id (speaker id is null because chunks can span turns of both speakers; this column is only present for ease of concatenation with the monologue version)
- *dynamic-time-warping-data.json*: dynamic time warped distance between the embeddings of the turns of each participant – this is raw distance, so it needs to be normalized by turn count before being used as a predictor variable
- *iafft-dbs.json*: contains the IAAFT surrogates for each conversation and the associated BDS statistics
- *individual-regime-chunk-data.csv*: same as *dyadic-regime-chunk-data.csv* but for individual speaker's semantic similarity trajectories
- *interleaved-data.json*: semantic similarity trajectories and PNR values for interleaved surrogate conversations; includes PNR values for both the original conversations each half was taken from, and for the individual halves from which the conversation is composed
- *interracial-friends-PCs.json*: for each conversation ID, includes pink noise robustness score (PNR), all covariates, and the first the first principal component of the three survey categories (Enjoyment, Connection, and Own traits)
- *latent-mutual-information.json*: mutual information scores between the embeddings of the turns of each participant
- monologue_breakpoint_shuffle.json: for each breakpoint-shuffled monologue (one participant's half of a conversation), the updated similarity trajectory, noise scaling coefficient, PNR value – and the original PNR value to compare against
- pnr-distance-matrix.csv: pairwise distances between conversations' PNR scores for the subset of conversations in which one of the participants took part in 5+ conversations
- user-distance-matrix.csv: pairwise distances between conversations based on user identity – the conversation receives a 1 if they share a speaker; a 0 if they don't

claude-dialogues contains:

- transcripts: A folder containing all 100 Claude-generated conversations, labeled with their condition (good or bad)
- *claude-conversation-pnrs.csv*: for each conversation, its condition, noise scaling coefficient (Hurst) and pink noise robustness score (PNR)
- *links-to-chats-with-claude.txt*: a text file with public links to the two conversations with Claude we used to generate the good and bad conversations
- *attention-checks.csv*: for each conversation, the category, order of creation, and 3 attention check questions – all with four potential answers, the correct answer listed first. These attention check questions were used to screen out Prolific participants who did not fully read the conversation before answering questions about enjoyment.
- *Original-prolific-data.csv*: this file contains all of the Prolific participants' responses to the attention check questions and enjoyment ratings of the Claude conversations. This format maintains all metadata, but is messy to work with – see *figures-and-statistics.ipynb* for the code we used to clean the data.

longitudinal-podcasts contains:

- audio-and-transcripts: for each podcast, this folder contains audio files and transcripts in both .srt and .txt format.
- podcast-pnrs: for each of the 25 podcasts, this folder contains a csv file with information on the first 50 episodes of the podcast: episode title, number, exclusion reason (if excluded), noise scaling coefficient (Hurst), pink noise robustness score (PNR), and covariates

speed-dating contains:

- blink-date:
  - transcripts of all dates from the Blink Date corpus
  - *blink-date-pnrs.csv*: for each date, whether the pair matched, the noise scaling coefficient (Hurst), and pink noise robustness score (PNR)

supplementary contains:

- *backbiter-data.json*: noise scaling coefficients, semantic similarity trajectories, and PNR values for backbiter transcripts of the CANDOR conversations (i.e., those with backchannels removed)
- *candor-iaaft-surrogates.zip*: IAFFT surrogates for each candor conversation, for the purpose of reproducing the BDS statistic results
- *convo2dfa.json*: detrended fluctuation analysis values for each chunk length in the original and interleaved CANDOR conversations; for the purpose of identifying where in frequency space power diverges
- *crqa-binarized-data.json*: recurrence and determinism values from standard CRQA analysis on CANDOR conversation turns
- *crqa-data.json*: recurrence and determinism values for a continuous version of CRQA on CANDOR conversation turns
- *e5_hursts.json*: scaling coefficients and PNR values for CANDOR conversations embedded with E5-large-v2
- *embeddings*: a folder containing MPNet embeddings for the Speed Dating corpus, Claude conversation scripts, and Longitudinal Podcasts corpus

- *gtr_hursts.json*: scaling coefficients and PNR values for CANDOR conversations embedded with GTR-T5-base
- *splade_hursts.json*: scaling coefficients and PNR values for CANDOR conversations embedded with splade-cocondenser-ensembledistil
- *word2vec_hursts.json*: scaling coefficients and PNR values for CANDOR conversations embedded with Word2Vec

Supplementary Models: specifications and results

1 Pink noise reflects dyadic structure beyond individual contributions

Model 1.0.1 – Individual PNRs < dyadic PNRs

$PNR \sim condition + (1|convo\ id)$

```
Fixed effects:
                      Estimate Std. Error         df t value Pr(>|t|)
(Intercept)            1.43356    0.02895 4920.94113   49.53   <2e-16 ***
conditionindividual   -0.86914    0.03427 3308.99990  -25.36   <2e-16 ***
```

Model 1.0.2 – Symmetric function predicting dyadic PNR from individual PNRs

$\bar{P}_{d_{ij}}$ = average of the two individual PNRs
Δ_{ij} = difference between the two individual PNRs (sign agnostic, since it's always squared)

$PNR \sim \bar{P}_{d_{ij}} + \Delta_{ij}^2 + \bar{P}_{d_{ij}}\Delta_{ij}^2 + \bar{P}_{d_{ij}}^3$

1.1 Metastable regime anlaysis

Model 1.1.1 – Relationship between regime chunk variance and mean similarity values

$chunk\ mean \sim chunk\ variance + chunk\ length + (1|convo\ id)$

```
Fixed effects:
              Estimate Std. Error         df t value Pr(>|t|)
(Intercept)  1.607e-01  6.435e-04  3.575e+03  249.76   <2e-16 ***
chunk_var    2.036e+00  2.095e-02  5.201e+04   97.18   <2e-16 ***
chunk_len   -9.313e-04  3.417e-05  5.170e+04  -27.25   <2e-16 ***
```

Model 1.1.2 – Individual/dyadic regime overlaps; state version

For each speaker, an overlap score (Hamming similarity) is calculated between the individual and the dyadic state trajectories. This overlap score is z-scored against a null constructed from an exhaustive circle shift procedure. Then, we test whether these z scores consistently exceed zero, accounting for the dependence introduced by each dyadic state trajectory being used twice, with a linear model of the form $overlap\ z\ score \sim 1 + (1|convo\ id)$

```
Fixed effects:
                Estimate Std. Error        df t value Pr(>|t|)
(Intercept)     2.273e-02  1.811e-02 1.654e+03   1.255     0.21
```

Model 1.1.3 – Individual/dyadic regime overlaps; continuous variance version

In case the median split was too coarse a measure to capture overlapping state structure between individual and dyadic variance trajectories, we additionally calculated Pearson correlation coefficients between each pair. Using the same circle shuffled z-scoring technique just described, we tested whether the variance correlation z-scores significantly exceeded 0 with a model of the form $variance\ correlation\ z\ score \sim 1 + (1|convo\ id)$. However, this model did not converge. After confirming that variance correlation z-scores were not correlated within conversation id, we dropped the random effect of conversation.

```
Coefficients:
            Estimate Std. Error t value Pr(>|t|)
(Intercept) -0.04373    0.01793   -2.44   0.0148 *
```

2 Pink noise predicts enjoyment and sense of connection during conversation

2.1 Controlling for basic conversation properties

Model 2.1.1 – Basic convo properties control; enjoyment

enjoyment $\sim pnr + mean\ turn\ duration + std\ turn\ duration + mean\ turn\ words + std\ turn\ words + backchannel\ rate + ASR\ proxy + duration + turn\ count$

```
Coefficients:
                    Estimate Std. Error t value Pr(>|t|)
(Intercept)       -0.0004183  0.0791421  -0.005   0.9958
pnr                0.1840507  0.0802800   2.293   0.0220 *
mean_turn_duration 0.3957709  0.2273100   1.741   0.0819 .
std_turn_duration -0.2240692  0.1361164  -1.646   0.0999 .
mean_turn_words    0.5279630  0.2277701   2.318   0.0206 *
std_turn_words     0.0478006  0.1327870   0.360   0.7189
backchannel_rate  -0.2311741  0.1065618  -2.169   0.0302 *
asr_proxy         -0.0800461  0.0830425  -0.964   0.3352
duration           0.2324025  0.2022141   1.149   0.2506
turn_count         1.1704508  0.2143356   5.461 5.46e-08 ***
```

Model 2.1.2 – Basic convo properties control; connection

connection $\sim pnr + mean\ turn\ duration + std\ turn\ duration + mean\ turn\ words + std\ turn\ words + backchannel\ rate + ASR\ proxy + duration + turn\ count$

```
Coefficients:
                    Estimate Std. Error t value Pr(>|t|)
(Intercept)       -0.0002924  0.0643836  -0.005  0.99638
pnr                0.1365517  0.0653093   2.091  0.03670 *
mean_turn_duration 0.1277255  0.1849209   0.691  0.48985
std_turn_duration -0.1017594  0.1107333  -0.919  0.35825
mean_turn_words    0.5265412  0.1852952   2.842  0.00454 **
std_turn_words    -0.0974133  0.1080247  -0.902  0.36731
backchannel_rate  -0.1079141  0.0866900  -1.245  0.21337
asr_proxy         -0.1078725  0.0675566  -1.597  0.11051
duration           0.1331480  0.1645049   0.809  0.41841
turn_count         0.7207317  0.1743660   4.133 3.75e-05 ***
```

2.2 Controlling for static and simple linear measures of similarity

Model 2.2.1 – Simple controls; enjoyment

$$\textbf{enjoyment} \sim pnr + average\ similarity + similarity\ variance + similarity\ slope$$

```
Coefficients:
                    Estimate Std. Error t value Pr(>|t|)
(Intercept)         -0.009027   0.083111  -0.109 0.913520
pnr                  0.288917   0.084702   3.411 0.000663 ***
average_similarity  -0.226580   0.130498  -1.736 0.082702 .
similarity_variance -0.413557   0.126646  -3.265 0.001115 **
similarity_slope     0.472270   0.114366   4.129 3.82e-05 ***
```

Model 2.2.2 – Simple controls; connection

$$\textbf{connection} \sim pnr + average\ similarity + similarity\ variance + similarity\ slope$$

```
Coefficients:
                    Estimate Std. Error t value Pr(>|t|)
(Intercept)         -0.00509    0.06575  -0.077 0.938301
pnr                  0.19225    0.06701   2.869 0.004169 **
average_similarity  -0.14079    0.10324  -1.364 0.172841
similarity_variance -0.38200    0.10019  -3.813 0.000142 ***
similarity_slope     0.26789    0.09047   2.961 0.003110 **
```

2.3 Controlling for more complex linear measures

Model 2.3.1 – PNR vs ARMA; enjoyment

$$\textbf{enjoyment} \sim pnr + AR + MA$$

```
Coefficients:
              Estimate Std. Error t value Pr(>|t|)
(Intercept) -0.0004183  0.0844885  -0.005  0.99605
pnr          0.2129486  0.1048658   2.031  0.04245 *
ar           1.6040218  0.5020028   3.195  0.00142 **
ma           1.5679000  0.4820199   3.253  0.00117 **
```

Model 2.3.2 – PNR vs ARMA; connection

$$\textbf{connection} \sim pnr + AR + MA$$

```
Coefficients:
              Estimate Std. Error t value Pr(>|t|)
(Intercept) -0.0002924  0.0668431  -0.004  0.99651
pnr          0.1528361  0.0829646   1.842  0.06563 .
ar           1.0247695  0.3971599   2.580  0.00996 **
ma           1.0133400  0.3813504   2.657  0.00795 **
```

Model 2.3.3 – PNR vs mutual information; enjoyment

$$\textbf{enjoyment} \sim pnr + LMI$$

```
Coefficients:
              Estimate Std. Error t value Pr(>|t|)
(Intercept) -0.0004183  0.0844508  -0.005 0.996049
pnr          0.3520590  0.0845225   4.165 3.27e-05 ***
lmi          0.2811657  0.0845225   3.327 0.000899 ***
```

Model 2.3.4 – PNR vs mutual information; connection

$$\textbf{connection} \sim pnr + LMI$$

```
Coefficients:
              Estimate Std. Error t value Pr(>|t|)
(Intercept) -0.0002924  0.0667647  -0.004 0.996506
pnr          0.2362888  0.0668214   3.536 0.000417 ***
lmi          0.2111583  0.0668214   3.160 0.001606 **
```

Model 2.3.5 – PNR vs dynamic time warping; enjoyment

$$\textbf{enjoyment} \sim pnr + DTW$$

```
Coefficients:
              Estimate Std. Error t value Pr(>|t|)
(Intercept) -0.0004183  0.0833184  -0.005 0.995995
pnr          0.2907181  0.0838805   3.466 0.000542 ***
dtw         -0.6309392  0.0838805  -7.522 8.82e-14 ***
```

Model 2.3.6 – PNR vs dynamic time warping; connection

$$\textbf{connection} \sim pnr + DTW$$

```
Coefficients:
              Estimate Std. Error t value Pr(>|t|)
(Intercept) -0.0002924  0.0662483  -0.004  0.99648
pnr          0.1987385  0.0666953   2.980  0.00293 **
dtw         -0.4001485  0.0666953  -6.000 2.42e-09 ***
```

Model 2.3.7 – PNR vs all complex linear measures; enjoyment

$$\textbf{enjoyment} \sim pnr + AR + MA + DTW + LMI$$

```
Coefficients:
              Estimate Std. Error t value Pr(>|t|)
(Intercept) -0.0004183  0.0826488  -0.005  0.99596
pnr          0.0871691  0.1040062   0.838  0.40209
ar           2.1444711  0.5002080   4.287 1.91e-05 ***
ma           2.1361988  0.4803217   4.447 9.27e-06 ***
dtw         -0.6887016  0.0845769  -8.143 7.53e-16 ***
lmi          0.2323648  0.0833433   2.788  0.00536 **
```

Model 2.3.8 – PNR vs all complex linear measures; connection

$$\textbf{connection} \sim pnr + AR + MA + DTW + LMI$$

```
Coefficients:
              Estimate Std. Error t value Pr(>|t|)
(Intercept) -0.0002924  0.0658723  -0.004 0.996459
pnr          0.0746192  0.0828944   0.900 0.368160
ar           1.3485016  0.3986732   3.382 0.000735 ***
ma           1.3582242  0.3828235   3.548 0.000399 ***
dtw         -0.4360792  0.0674090  -6.469  1.3e-10 ***
lmi          0.1813145  0.0664258   2.730 0.006409 **
```

Model 2.3.9 – PNR vs DTW and lag-1 ACF; enjoyment

enjoyment $\sim pnr + DTW + lag1ACF$

```
Coefficients:
              Estimate Std. Error t value Pr(>|t|)
(Intercept) -0.0004183  0.0827255  -0.005    0.996
pnr          0.0550858  0.0958037   0.575    0.565
dtw         -0.7016194  0.0844861  -8.305  < 2e-16 ***
lag1_acf     0.4755340  0.0955613   4.976 7.16e-07 ***
```

Model 2.3.10 – PNR vs ARMA and lag-1 ACF; enjoyment

enjoyment $\sim pnr + AR + MA + lag1ACF$

```
Coefficients:
              Estimate Std. Error t value Pr(>|t|)
(Intercept) -0.0004183  0.0844611  -0.005   0.9960
pnr          0.1997285  0.1052334   1.898   0.0579 .
ar          -0.0904979  1.2797972  -0.071   0.9436
ma          -0.0906557  1.2490079  -0.073   0.9421
lag1_acf     0.3588246  0.2493005   1.439   0.1502
```

2.4 Dyadic PNR explains variance in enjoyment and connection beyond individual PNRs

Model 2.4.1 – Individual vs dyadic PNR; enjoyment

own enjoyment $\sim pnr + pnr\ self + pnr\ other + (1|convo\ id)$

```
Fixed effects:
              Estimate Std. Error         df t value Pr(>|t|)
(Intercept)   -0.47087    0.10373 1652.00001  -4.540 6.04e-06 ***
pnr            0.20213    0.05173 1652.00001   3.908 9.69e-05 ***
pnr_self       0.14987    0.05002 3188.58875   2.996 0.002758 **
pnr_other      0.17039    0.05002 3188.58875   3.406 0.000667 ***
```

Model 2.4.2 – Individual vs dyadic PNR; connection

own connection $\sim pnr + pnr\ self + pnr\ other + (1|convo\ id)$

```
Fixed effects:
              Estimate Std. Error         df t value Pr(>|t|)
(Intercept)   -0.27858    0.08250 1652.00002  -3.377 0.000750 ***
pnr            0.14098    0.04114 1652.00002   3.427 0.000626 ***
pnr_self       0.07345    0.04039 3226.76745   1.818 0.069083 .
```

```
pnr_other        0.06159    0.04039 3226.76745   1.525 0.127367
```

To further separate the dyad-level signal from the contributions of the two individuals, we reparameterized the two individual terms into an across-dyad component (the pair's average individual PNR) and a within-dyad component (the difference between a participant's PNR and their partner's). Under this parameterization, the dyadic-PNR coefficient indexes the portion of dyadic PNR not linearly reducible to the two individuals; the average-PNR coefficient reflects the pair's mean individual level — a dyadic property in its own right, even though it is built from individual signals; and the within-dyad difference carries very little weight, indicating that predictive value attaches to the pair's joint level rather than to any asymmetry between partners.

Model 2.4.3 – Individual vs dyadic PNR re-parameterized; enjoyment

$$\textbf{own enjoyment} \sim pnr + average\ individual\ pnr + self\ pnr\ minus\ other + (1|convo\ id)$$

```
Fixed effects:
                      Estimate Std. Error         df t value Pr(>|t|)    
(Intercept)           -0.47087    0.10373 1652.00001  -4.540 6.04e-06 ***
pnr                    0.20213    0.05173 1652.00001   3.908 9.69e-05 ***
average_indiv_pnr      0.32026    0.07722 1652.00001   4.148 3.53e-05 ***
self_pnr_minus_other  -0.01026    0.03181 1653.99997  -0.323    0.747    
```

Model 2.4.4 – Individual vs dyadic PNR re-parameterized; connection

$$\textbf{own connection} \sim pnr + average\ individual\ pnr + self\ pnr\ minus\ other + (1|convo\ id)$$

```
Fixed effects:
                      Estimate Std. Error         df t value Pr(>|t|)    
(Intercept)         -2.786e-01  8.250e-02  1.652e+03  -3.377 0.000750 ***
pnr                  1.410e-01  4.114e-02  1.652e+03   3.427 0.000626 ***
average_indiv_pnr    1.350e-01  6.141e-02  1.652e+03   2.199 0.028032 *  
self_pnr_minus_other 5.927e-03  2.624e-02  1.654e+03   0.226 0.821283    
```

3 Replication in Interracial Friends corpus

Model 3.0.1 – Convo length controls; enjoyment

$$\textbf{enjoyment} \sim pnr + duration + turn\ count$$

```
Coefficients:
              Estimate Std. Error t value Pr(>|t|)   
(Intercept) -1.0578012  0.3886162  -2.722  0.00782 **
pnr          0.1708456  0.1653582   1.033  0.30435   
duration     0.0005222  0.0004114   1.269  0.20762   
turn_count   0.0030269  0.0017176   1.762  0.08150 . 
```

Model 3.0.2 – Convo length controls; connection

$$\textbf{connection} \sim pnr + duration + turn\ count$$

```
Coefficients:
              Estimate Std. Error t value Pr(>|t|)  
(Intercept) -0.6527546  0.3476253  -1.878   0.0637 .
```

```
pnr          0.1544775  0.1479163   1.044   0.2992
duration     0.0005100  0.0003680   1.386   0.1692
turn_count   0.0003531  0.0015365   0.230   0.8188
```

Model 3.0.3 – Basic convo properties control; enjoyment (IF replication)

enjoyment $\sim pnr + mean\ turn\ duration + std\ turn\ duration + mean\ turn\ words + std\ turn\ words + backchannel\ rate + ASR\ proxy$

```
Coefficients:
                   Estimate Std. Error t value Pr(>|t|)
(Intercept)        -7.75475    6.56675  -1.181   0.2411
pnr                 0.37605    0.17056   2.205   0.0303 *
mean_turn_duration -0.57369    0.35743  -1.605   0.1123
std_turn_duration   0.27131    0.23031   1.178   0.2422
mean_turn_words     0.17500    0.10290   1.701   0.0928 .
std_turn_words     -0.08051    0.06489  -1.241   0.2182
asr_proxy           8.36616    7.24429   1.155   0.2515
```

Model 3.0.4 – Basic convo properties control; connection (IF replication)

connection $\sim pnr + mean\ turn\ duration + std\ turn\ duration + mean\ turn\ words + std\ turn\ words + backchannel\ rate + ASR\ proxy$

```
Coefficients:
                   Estimate Std. Error t value Pr(>|t|)
(Intercept)        -0.33375    5.80603  -0.057    0.954
pnr                 0.25055    0.15080   1.661    0.100
mean_turn_duration -0.13852    0.31602  -0.438    0.662
std_turn_duration   0.06368    0.20363   0.313    0.755
mean_turn_words     0.04995    0.09098   0.549    0.584
std_turn_words     -0.02822    0.05737  -0.492    0.624
asr_proxy           0.36863    6.40508   0.058    0.954
```

4 Pink noise predicts real-world behavioral outcomes during speed dates

Model 4.0.1 – Predicting first date outcomes from PNR

$glm(matched \sim pnr + duration + turn\ count, family = binomial(link = "logit")$ Because this is a one-sided test, we can run $glht(model, linfct = c("pnr <= 0"))$

```
Linear Hypotheses:
             Estimate Std. Error z value Pr(>z)
pnr <= 0     0.7007     0.4158   1.685  0.046 *
```

5 Pairs' repeated conversations demonstrate stronger pink noise over time

Model 5.0.1 – Predicting PNR from episode number with controls

$pnr\ episode\ number + mean\ turn\ len + std\ turn\ len + mean\ turn\ words + std\ turn\ words + asr\ proxy + duration + turn\ count + (1|podcast)$

Fixed effects:

	Estimate	Std. Error	df	t value	Pr(>|t|)	
(Intercept)	1.531e+00	2.240e+00	6.432e+01	0.684	0.4967	
episode_number	5.551e-03	2.656e-03	9.993e+02	2.090	0.0369	*
mean_turn_len	-1.083e-01	7.675e-02	1.638e+02	-1.411	0.1602	
std_turn_len	7.441e-02	4.514e-02	3.658e+02	1.648	0.1001	
mean_turn_words	3.418e-02	2.692e-02	1.279e+02	1.270	0.2065	
std_turn_words	-2.522e-02	1.597e-02	2.508e+02	-1.579	0.1156	
asr_proxy	-8.036e-01	2.434e+00	6.689e+01	-0.330	0.7423	
duration	3.497e-05	4.912e-05	6.562e+01	0.712	0.4790	
turn_count	1.116e-03	4.428e-04	7.048e+01	2.519	0.0140	*

REFERENCES

1. E. E. Levine, A. R. Roberts, T. R. Cohen, Difficult conversations: Navigating the tension between honesty and benevolence. *Curr. Opin. Psychol.* **31**, 38–43 (2020).

2. P. Dao, Effects of task goal orientation on learner engagement in task performance. *IRAL Int. Rev. Appl. Linguist. Lang. Teach.* **59**, 315–334 (2021).

3. C. N. Wright, Educational orientation and upward influence: An examination of students' conversations about disappointing grades. *Commun. Educ.* **61**, 271–289 (2012).

4. E. M. Templeton, L. J. Chang, E. A. Reynolds, M. D. Cone LeBeaumont, T. Wheatley, Fast response times signal social connection in conversation. *Proc. Natl. Acad. Sci. U.S.A.* **119**, e2116915119 (2022).

5. A. Reece, G. Cooney, P. Bull, C. Chung, B. Dawson, C. Fitzpatrick, T. Glazer, D. Knox, A. Liebscher, S. Marin, The CANDOR corpus: Insights from a large multimodal dataset of naturalistic conversation. *Sci. Adv.* **9**, eadf3197 (2023).

6. D. A. Coker, J. Burgoon, The nature of conversational involvement and nonverbal encoding patterns. *Hum. Commun. Res.* **13**, 463–494 (1987).

7. M. G. Frank, P. Ekman, W. V. Friesen, Behavioral markers and recognizability of the smile of enjoyment. *J. Pers. Soc. Psychol.* **64**, 83–93 (1993).

8. A. S. Gabay, A. Pisauro, K. C. O'Nell, M. A. J. Apps, Social environment-based opportunity costs dictate when people leave social interactions. *Commun. Psychol.* **2**, 42 (2024).

9. S. Tsang, K. Barrentine, S. Chadha, S. Oishi, A. Wood, Social exploration: How and why people seek new connections. *Psychol. Rev.* **132**, 656–679 (2024).

10. C. Geishauser, C. van Niekerk, H. Lin, N. Lubis, M. Heck, S. Feng, M. Gašić, "Dynamic dialogue policy for continual reinforcement learning" in *Proceedings of the 29th International Conference on Computational Linguistics*, N. Calzolari, C.-R. Huang, H. Kim, J. Pustejovsky, L. Wanner, K.-S. Choi, P.-M. Ryu, H.-H. Chen, L. Donatelli, H. Ji, S.

Kurohashi, P. Paggio, N. Xue, S. Kim, Y. Hahm, Z. He, T. K. Lee, E. Santus, F. Bond, S.-H. Na, Eds. (International Committee on Computational Linguistics, 2022), pp. 266–284; https://aclanthology.org/2022.coling-1.21.

11. P. A. Heeman, "Representing the Reinforcement Learning state in a negotiation dialogue" in *2009 IEEE Workshop on Automatic Speech Recognition & Understanding* (2009), pp. 450–455; https://ieeexplore.ieee.org/document/5373413.

12. M. Gallotti, C. D. Frith, Social cognition in the we-mode. *Trends Cogn. Sci.* **17**, 160–165 (2013).

13. M. E. Ireland, R. B. Slatcher, P. W. Eastwick, L. E. Scissors, E. J. Finkel, J. W. Pennebaker, Language style matching predicts relationship initiation and stability. *Psychol. Sci.* **22**, 39–44 (2011).

14. A. L. Gonzales, J. T. Hancock, J. W. Pennebaker, Language style matching as a predictor of social dynamics in small groups. *Commun. Res.* **37**, 3–19 (2010).

15. C.-W. Yu, Y.-S. Chuang, A. N. Lotsos, T. Meier, C. M. Haase, The more similar, the better? Associations between latent semantic similarity and emotional experiences differ across conversation contexts. *J. Lang. Soc. Psychol.* **44**, 961–984 (2025).

16. P. J. Taylor, S. Thomas, Linguistic style matching and negotiation outcome. *Negot. Confl. Manag. Res.* **1**, 263–281 (2008).

17. R. Dale, M. J. Spivey, Unraveling the Dyad: Using recurrence analysis to explore patterns of syntactic coordination between children and caregivers in conversation. *Lang. Learn.* **56**, 391–430 (2006).

18. R. Fusaroli, K. Tylén, Investigating conversational dynamics: Interactive alignment, interpersonal synergy, and collective task performance. *Cognit. Sci.* **40**, 145–171 (2016).

19. D. H. Abney, A. Paxton, R. Dale, C. T. Kello, Complexity matching in dyadic conversation. *J. Exp. Psychol. Gen.* **143**, 2304–2315 (2014).

20. M. T. Tolston, M. A. Riley, V. Mancuso, V. Finomore, G. J. Funke, Beyond frequency counts: Novel conceptual recurrence analysis metrics to index semantic coordination in team communications. *Behav. Res. Methods* **51**, 342–360 (2019).

21. C.-K. Peng, S. V. Buldyrev, S. Havlin, M. Simons, H. E. Stanley, A. L. Goldberger, Mosaic organization of DNA nucleotides. *Phys. Rev. E* **49**, 1685–1689 (1994).

22. L. Rydin Gorjão, G. Hassan, J. Kurths, D. Witthaut, MFDFA: Efficient multifractal detrended fluctuation analysis in python. *Comput. Phys. Commun.* **273**, 108254 (2022).

23. G. C. Van Orden, H. Kloos, S. Wallot, "Living in the pink: Intentionality, wellbeing, and complexity" in *Philosophy of Complex Systems*, C. Hooker, Ed. (Elsevier, 2011), pp. 629–672; www.sciencedirect.com/science/article/pii/B9780444520760500225.

24. Anthropic, Claude 3.7 Sonnet; www.anthropic.com/claude/sonnet.

25. D. Angus, Recurrence methods for communication data, reflecting on 20 years of progress. *Front. Appl. Math. Stat.* **5**, 54 (2019).

26. U. Hasson, C. D. Frith, Mirroring and beyond: Coupled dynamics as a generalized framework for modelling social interactions. *Philos. Trans. R. Soc. Lond. B Biol. Sci.* **371**, 20150366 (2016).

27. T. Wheatley, M. A. Thornton, A. Stolk, L. J. Chang, The emerging science of interacting minds. *Perspect. Psychol. Sci.* **19**, 355–373 (2024).

28. R. Fusaroli, J. Rączaszek-Leonardi, K. Tylén, Dialog as interpersonal synergy. *New Ideas Psychol.* **32**, 147–157 (2014).

29. E. Tognoli, M. Zhang, A. Fuchs, C. Beetle, J. A. S. Kelso, Coordination dynamics: A foundation for understanding social behavior. *Front. Hum. Neurosci.* **14**, 317 (2020).

30. I. Ravreby, Y. Shilat, Y. Yeshurun, Liking as a balance between synchronization, complexity and novelty. *Sci. Rep.* **12**, 3181 (2022).

31. J. Michalsky, H. Schoormann, "Pitch convergence as an effect of perceived attractiveness and likability" (2017), pp. 2253–2256; www.isca-archive.org/interspeech_2017/michalsky17_interspeech.html.

32. J. L. Lakin, T. L. Chartrand, Using nonconscious behavioral mimicry to create affiliation and rapport. *Psychol. Sci.* **14**, 334–339 (2003).

33. S. E. Brennan, H. H. Clark, Conceptual pacts and lexical choice in conversation. *J. Exp. Psychol. Learn. Mem. Cogn.* **22**, 1482–1493 (1996).

34. S. E. Brennan, Lexical entrainment in spontaneous dialog (1996).

35. C. Welker, T. Wheatley, G. Cason, C. Gorman, M. Meyer, Self-views converge during enjoyable conversations. *Proc. Natl. Acad. Sci. U.S.A.* **121**, e2321652121 (2024).

36. K. Song, X. Tan, T. Qin, J. Lu, T.-Y. Liu, MPNet: Masked and permuted pre-training for language understanding. arXiv:2004.09297 [cs.CL] (2020).

37. HuggingFace, all-mpnet-base-v2 Model Card (2024); https://huggingface.co/sentence-transformers/all-mpnet-base-v2.

38. N. Reimers, I. Gurevych, Sentence-BERT: Sentence embeddings using Siamese BERT-Networks. arXiv:1908.10084 [cs.CL] (2019).

39. K. A. Yuksel, T. Ferreira, A. Gunduz, M. Al-Badrashiny, G. Javadi, "A reference-less quality metric for automatic speech recognition via contrastive-learning of a multi-language model with self-supervision" in *2023 IEEE International Conference on Acoustics, Speech, and Signal Processing Workshops (ICASSPW)* (IEEE, 2023), pp. 1–5; https://ieeexplore.ieee.org/document/10193003.

40. R. Tavenard, J. Faouzi, G. Vandewiele, F. Divo, G. Androz, C. Holtz, M. Payne, R. Yurchak, M. Rußwurm, K. Kolar, E. Woods, Tslearn, a machine learning toolkit for time series data. *J. Mach. Learn. Res.* **21**, 1–6 (2020).

41. M. Bain, J. Huh, T. Han, A. Zisserman, “WhisperX: Time-accurate speech transcription of long-form audio” (2023), pp. 4489–4493; www.isca-archive.org/interspeech_2023/bain23_interspeech.html.

42. M. Ashraf, Whisper Diarization: Speaker Diarization Using OpenAI Whisper (2024); https://github.com/MahmoudAshraf97/whisper-diarization.

43. R. Fusaroli, K. Tylén, K. Garly, J. Steensig, M. H. Christiansen, M. Dingemanse, “Measures and mechanisms of common ground: Backchannels, conversational repair, and interactive alignment in free and task-oriented social interactions” in *Proceedings of the 39th Annual Conference of the Cognitive Science Society* (Cognitive Science Society, 2017), pp. 2055–2060.

44. M. Dingemanse, N. J. Enfield, Interactive repair and the foundations of language. *Trends Cogn. Sci.* **28**, 30–42 (2024).

45. C. A. Bergey, S. DeDeo, From “um” to “yeah”: Producing, predicting, and regulating information flow in human conversation. arXiv:2403.08890 [cs.CL] (2024).

46. J. Ni, C. Qu, J. Lu, Z. Dai, G. H. Ábrego, J. Ma, V. Y. Zhao, Y. Luan, K. B. Hall, M.-W. Chang, Y. Yang, Large dual encoders are generalizable retrievers. arXiv:2112.07899 [cs.IR] (2021).

47. L. Wang, N. Yang, X. Huang, B. Jiao, L. Yang, D. Jiang, R. Majumder, F. Wei, Text embeddings by weakly-supervised contrastive pre-training. arXiv:2212.03533 [cs.CL] (2024).

48. T. Formal, C. Lassance, B. Piwowarski, S. Clcinchant, From distillation to hard negative sampling: Making sparse neural IR models more effective. arXiv:2205.04733 [cs.IR] (2022).

49. T. Mikolov, K. Chen, G. Corrado, J. Dean, Efficient estimation of word representations in vector space. arXiv:1301.3781 [cs.CL] (2013).

50. B. M. Goldstein, S. M. Burns, F. C. Peck, R. Dale, M. D. Lieberman, Cross-recurrence quantification analysis captures inter-brain coupling during naturalistic negotiation: A new dynamic approach for hyperscanning. *Front. Neurosci.* **19**, 1713357 (2025).
